# Genomic global epidemiology and evolution of Escherichia coli ST131 clade A

**DOI:** 10.1099/mgen.0.001858

**Published:** 2026-10-07

**Authors:** Yasufumi Matsumura, Gisele Peirano, Pierre Bogaerts, Te-Din Huang, Bogdan I. Iorga, Miki Nagao, Johann D.D. Pitout

**Affiliations:** 1 Department of Clinical Laboratory Medicine, Kyoto University Graduate School of Medicine, Kyoto, Japan; 2 Alberta Precision Laboratories, Calgary, Alberta, Canada; 3 Cumming School of Medicine, University of Calgary, Calgary, Alberta, Canada; 4 Laboratory of Microbiology, National Reference Center for Antimicrobial Resistance in Gram-negative Bacteria, CHU UCL Namur, Yvoir, Belgium; 5 Institut de Chimie des Substances Naturelles, CNRS UPR 2301, Université Paris-Saclay, Gif-sur-Yvette, France; 6 University of Pretoria, Pretoria, Gauteng, South Africa

**Keywords:** clade A, *Escherichia coli *ST131, genomic epidemiology

## Abstract

During the 2010s, *Escherichia coli* ST131 clade A tested sensitive to most antibiotics. In the 2020s, Europe experienced outbreaks of fluoroquinolone-resistant ST131-A that contained CTX-M β-lactamases and OXA-48-like carbapenemases. We investigated the acquisition of different antimicrobial resistance determinants in the emergence of multidrug-resistant (MDR) ST131-A. Phylogenetic analysis and Bayesian inference were utilized to define subclades and clusters (within subclades) and investigate the evolutionary history of ST131-A, using sequenced genomes and publicly available genomic data (*n*=2,905). ST131-A consisted of five dominant subclades and ten frequent clusters, representing 93 and 83% of the total population, respectively. The sampled most recent common ancestors of the dominant subclades dated mainly to the 1990s. Several MDR clusters linked with diverse quinolone resistance-determining region mutations, CTX-M and OXA-48-like genes diversified during the 2000s. The distribution and mobile genetic elements linked with *bla*_CTX-M-14_, *bla*_CTX-M-15_ and *bla*_CTX-M-27_ differed among ST131-A subclades and clusters across geographic locations. Three ST131-A clusters belonging to different subclades and carrying *bla*_OXA-48_ and *bla*_OXA-244_ in distinct genetic contexts were associated with the recent European dissemination. The identification of MDR ST131-A subclades and clusters with divergent molecular architectures emerging independently across regions highlights the challenges for public health surveillance and control efforts.

**Impact Statement**

While *Escherichia coli* ST131 clade C drove the initial global expansion of fluoroquinolone resistance and CTX-M enzymes, our findings show that clade A has independently diversified into multiple multidrug-resistant (MDR) subclades and distinct clusters since the 1990s. Recent European outbreaks reflect parallel but distinct evolutionary pathways, with heterogeneous mobile genetic elements carrying CTX-M and OXA-48-like genes across geographic regions. The identification of MDR ST131-A subclades with divergent molecular architectures emerging independently across regions highlights the challenges for public health surveillance and control efforts.

## Data Summary

All genome sequence data analysed in this study are publicly available. Accession numbers for all isolates are provided in Dataset S1. Of these, 96 isolates were newly sequenced as part of the present study and have been deposited in a public sequence repository (Dataset S1, available with the inline version of this article). All genome assemblies generated in this study are available in Zenodo at https://doi.org/10.5281/zenodo.18920691. Analysis scripts and workflow documentation used in this study are available at GitHub: https://github.com/yazblood/ST131A-genomics/.

## Introduction

*Escherichia coli* ST131 is one of the most successful multidrug-resistant (MDR) high-risk clones identified to date [1]. It was primarily responsible for the global increase of fluoroquinolone and cephalosporin resistance among *E. coli* during the mid to late 2000s [2]. ST131 is currently the most widespread clone among human *E. coli* and causes thousands of human infections and deaths annually [3, 4]. ST131 belongs to three clades (i.e. A, B, C) and four C subclades (i.e. C0, C1, C1-M27 and C2) [5]. The ST131 C subclades (specifically C1, C1-M27 and C2) are fluoroquinolone-resistant and currently dominate the ST131 population structure, accounting for up to 80% of global ST131 isolates [6]. ST131-C was crucial in the appearance and global expansion of CTX-M enzymes during the mid-2000s and early 2010s [3, 7]. ST131-C is also an important carbapenemase-producing clone that consisted mainly of KPCs (linked with ST131-C1) and NDMs (linked with ST131-C2) [8].

Overall, ST131-A is less frequently reported than the C subclades but remains an important cause of bloodstream infections (BSIs) in regions with low antibiotic consumption (such as Norway) [9]. In Calgary, Canada, population-based surveillance of *E. coli* BSIs over an 11 year period showed that ST131-A accounted for 10% of the overall ST131 population and increased in incidence from 2006 (0/100,000 residents) to 2016 (1.15/100,000 residents) [10]. ST131-A is the most common ST131 clade obtained from Calgary wastewater [11]. Until the 2020s, ST131-A remained, for the most part, sensitive to the fluoroquinolones and cephalosporins and β-lactamases were rare among this clade [4]. This scenario changed dramatically in the early 2020s with European community outbreaks of fluoroquinolone-resistant ST131-A (that included the single-locus ST131 variant named ST13730) that contained CTX-M β-lactamases and OXA-48-like carbapenemases [12, 13].

We performed phylogenetic analysis to define subclades and clusters within global ST131-A and investigated the evolutionary history of this clade using publicly available genomic data and genomes sequenced for this study. We investigated the acquisition of different antimicrobial resistance (AMR) determinants, specifically the quinolone resistance-determining region (QRDR) mutations, CTX-M and OXA-48-like genes, over time by MDR ST131-A subclades and clusters.

## Methods

### Bacterial isolates

We included 2,809 publicly available ST131-A genomes. This included single-locus ST131 variants, namely ST13730 and others (details in Table 1). All complete and draft *E. coli* genomes with short-read sequencing data were retrieved from the NCBI Assembly (*n*=153) and AllTheBacteria databases (August 2024 update; *n*=2,335) [14], which comprehensively cover the NCBI Sequence Read Archive, respectively. Additionally, ST131-A genomes (including single-locus variants) with short-read data from previous studies not included in the AllTheBacteria databases and were deposited in the NCBI Sequence database from previous studies (*n*=321) [9, 11, 12, 15–18] were also incorporated. Sequenced Japanese clinical surveillance isolates (*n*=86) obtained during 2004–2021 were also included, as well as clinical surveillance isolates (*n*=10) from Belgium obtained between 2023–2025. Only genomes with available metadata, including year and country of isolation, were analysed (Dataset S1). The genome selection process and available metadata for excluded genomes are summarized in Table S1 (available with the inline version of this article).

**Table 1.** Epidemiological and typing characteristics of dominant subclades among global *E. coli* ST131 clade A

	*A4 *n*=357 **(12%)**	A5 *n*=840 **(29%)**	A6 *n*=567 **(20%)**	A7 *n*=543 **(19%)**	A9 *n*=396 **(14%)**	All *n*=2,905 **(100%)**
Sequence type:						
ST131	353 (99%)	803 (96%)†	567 (100%)†	538 (99%)	390 (98%)	2,852 (98%)
ST13730	0	34 (4%)†	0^†^	0†	0	34 (1%)
Other STs‡	4 (1%)	3 (<1%)	0	5 (1%)	6 (2%)	19 (<1%)
Clusters:*						
Total number of clusters	2	1	3	11	12	79
Most frequent clusters	16_A4 (*n*=196 [55%])	21_A5 (*n*=840 [100%])	19_A6 (*n*=489 [86%])	49_A7 (*n*=205 [38%])	33_A9 (*n*=98 [25%])	21_A5 (*n*=840 [29%])
	17_A4 (*n*=161 [45%])		18_A6 (*n*=75 [13%])	47_A7 (*n*=150 [28%])	29_A9 (*n*=66 [17%])	19_A6 (*n*=489 [17%])
				48_A7 (*n*=119 [22%])		
Geographic location:						
Americas	82 (23%)	270 (32%)†	68 (12%)†	157 (29%)†	45 (11%)†	643 (22%)
Asia/Pacific	193 (54%)†	145 (17%)†	326 (57%)†	132 (24%)†	146 (37%)	1,042 (36%)
Europe/UK	72 (20%)†	392 (47%)†	147 (26%)†	227 (42%)	198 (50%)†	1,116 (38%)
Other	10 (3%)	33 (4%)	26 (5%)	27 (5%)	7 (2%)	104 (4%)
Reporting period:						
Pre-2010	17 (5%)	7 (1%)†	0†	7 (1%)†	30 (8%)†	98 (3%)
2010–2018	242 (68%)†	473 (56%)†	232 (41%)†	179 (33%)†	240 (61%)†	1,453 (50%)
Post-2018	98 (27%)†	360 (43%)†	335 (59%)†	357 (66%)†	126 (32%)†	1,354 (47%)
FimH:						
FimH41	327 (92%)	814 (97%)†	456 (80%)†	527 (97%)†	376 (95%)	2,684 (92%)
FimH89	1 (<1%)†	0†	79 (14%)†	0†	1 (<1%)†	81 (3%)
FimH other	27 (8%)†	24 (3%)†	28 (5%)	14 (3%)	17 (4%)	128 (4%)
FimH neg	2 (<1%)	2 (<1%)	4 (1%)	2 (<1%)	2 (<1%)	12 (<1%)
O-type:						
O16	354 (99%)†	814 (97%)†	565 (100%)†	536 (99%)†	302 (76%)†	2,708 (93%)
O25b	0†	0†	1 (<1%)†	1 (<1%)†	36 (9%)†	80 (3%)
O-type others	1 (<1%)†	20 (2%)	0†	4 (<1%)†	40 (10%)†	83 (3%)
Untypeable	2 (<1%)	6 (<1%)	1 (<1%)†	2 (<1%)	18 (5%)†	34 (1%)
H-type:						
H5	355 (99%)†	834 (99%)†	566 (100%)†	537 (99%)†	358 (90%)†	2,797 (96%)
H4	0†	0†	0†	1 (<1%)†	37 (9%)†	93 (3%)
Untypeable	1 (<1%)	1 (<1%)	1 (<1%)	0	0	3 (<1%)

*ST131-A subclades were defined at the first BAPS level clustering. Clusters within the different subclades were defined at the second BAPS level clustering.

†Bonferroni-corrected *P*<0.05 by Fisher’s exact test, comparing each subclade with all remaining ST131-A genomes

‡Unassigned single-locus variants of ST131 (*n*=3), ST11362 (*n*=2), ST13704 (*n*=2), ST14587 (*n*=2) and one each of ST1982, ST2639, ST2784, ST5896, ST7214, ST8420, ST17122, ST17197 and ST17198.

### Genomic analysis

Short read sequencing libraries were prepared using the Illumina DNA prep kit (Illumina, San Diego, CA, USA) and sequenced on an Illumina NextSeq 1,000 for 300 cycles (151 bp paired-end). Long-read sequencing libraries were prepared using the Rapid Sequencing DNA V14 kit (SQK-RBK114.24) and the Ligation Sequencing Kit (SQK-LSK109) and were analysed with PromethION2 Solo or GridION with R10.4.1 flow cells or a MinION with R9.4.1 flow cells (Oxford Nanopore Technologies, Oxford, UK).

Draft genomes were assembled using SPAdes v3.15.5. For isolates with both long- and short-read data, hybrid assemblies were generated using autocycler v.0.5.2 [19] or a combination of flye v.2.9.2 [20], medaka v.1.11.1 (https://github.com/nanoporetech/medaka) and polypolish v.0.5.0 [21] (Dataset S2). Genomes were retained if they met the following quality control criteria: sequencing depth >30×, N50 >30 kb, <500 contigs and CheckM2 (v.1.0.2) estimates of genomic contamination of <5% and completeness of >90% [22]. Multilocus sequence typing (MLST) was performed using the mlst v.2.23.0 software (https://github.com/tseemann/mlst) according to public schemes [23]. ST131 clades were determined according to the phylogeny and reference genomes for each clade (Dataset S1).

AMR genes and AMR chromosomal mutations were detected using AMRFinderPlus v.4.0.23 (database version 2025-07-16.1) [24]. Virulence-associated factors (VAFs) were defined using VirulenceFinder v.3.1.0 [25]. Plasmids were typed using PlasmidFinder v.2.1.0 [26], plasmid MLST scheme [23] and MOB-suite v.3.1.9 [27]. Complete plasmid sequences were analysed using Pling v.3.0.2, which combines sequence-containment and double-cut-and-join indel rearrangement distances to construct a plasmid relatedness network and identify communities and subcommunities [28]. To determine plasmid similarities, sequences were compared using blast [29] and coverage and identity values were calculated.

A core SNP-based phylogenetic tree was constructed by mapping short reads against the ST131 clade A strain MVAST0167 (GenBank Accession no. GCA_001566655.1) using snippy v4.6.0 (https://github.com/tseemann/snippy). We used core SNPs that were present in >95% of genomes. Recombination regions determined by Gubbins v3.425 [30] were excluded and a maximum-likelihood tree was created using IQ-TREE 2 v2.4.0 under the TVM+F+I+R5 model selected by ModelFinder [31]. Branch support was assessed using the Shimodaira–Hasegawa-like approximate likelihood ratio test (SH-aLRT) and ultrafast bootstrap (UFBoot), each with 1,000 replicates. The recommended IQ-TREE thresholds of SH-aLRT ≥80% and UFBoot ≥95% were used to summarize whether nodes met both support criteria. Root-to-tip regression was performed using TempEst v.1.5.3 [32] on the ST131-A-only phylogeny, with the best-fitting root selected by maximizing R². The hierarchical Bayesian analysis of population structure (BAPS) clustering was performed using fastbaps, which approximates a Dirichlet process mixture model for multilocus genotype data and identifies clusters by maximizing the model marginal likelihood [33]. The optimized.baps prior hyperparameter was used to define the ST131-A population structure, with the first- and second-level partitions designated as subclades and clusters, respectively. Sensitivity to prior specification was assessed by repeating clustering with the other three available fastbaps prior settings. The stability of the inferred fastbaps clusters was assessed using bootstrap co-clustering analysis. We used Bayesian inference (BEAST v.2.7.7) to infer the time-scaled phylogeny [34]. We used general time-reversible substitution, a strict or optimized relaxed molecular clock and Bayesian skyline population models. To assess clock-model fit, we compared the strict and relaxed molecular clock models using path-sampling marginal likelihood estimation in BEAST. Bayes factors were calculated from differences in log marginal likelihoods between models. For each clock model, three independent Markov chain Monte Carlo runs were performed and extended as necessary until convergence was achieved. Convergence was assessed by visual inspection of trace plots and by confirming that the effective sample size for all parameters were >200. A maximum clade credibility tree with median heights was generated from the combined posterior tree sample using TreeAnnotator v.2.7.7. ‘Dominant’ subclades were operationally defined as subclades that consisted of >10% of the total population [35]. This pragmatic cutoff was used to focus the comparative analyses on the most abundant lineages and was not intended to represent a biological boundary.

### Statistical analysis

Differences in characteristics across dominant subclades were evaluated using Fisher’s exact test. Subclade- and cluster-specific differences were assessed by comparing each subclade or cluster with all remaining ST131 genomes with Bonferroni correction. *P*-values <0.05 were considered statistically significant. Statistical analyses were performed using the stats package in R software version 4.4.2 (https://cran.r-project.org).

## Results

### ST131 clade A consisted of five dominant subclades and ten frequent clusters

We included a total of 2,905 ST131-A genomes, including 178 complete genomes and 2,727 draft genomes. This included the following STs: ST131-A (*n*=2,852) and ST131-A single-locus variants, such as ST13730 (*n*=34) and others (*n*=19) (Table 1). BAPS analysis of the 2,905 genomes divided ST131-A into 22 subclades (A1–A22) at the first level of BAPS clustering (Fig. 1 and S1). The numbers and proportions of genomes assigned to the 22 subclades are shown in Table S2. ST13730 (*n*=34) genomes clustered within ST131-A5. Subclades A4 (12%), A5 (29%), A6 (20%), A7 (19%) and A9 (14%) were dominant (i.e. each representing >10% of the total population) [Table 1]. The five dominant subclades represented 93% of the total ST131-A population and were included in the tables for analysis.

**Fig. 1.**
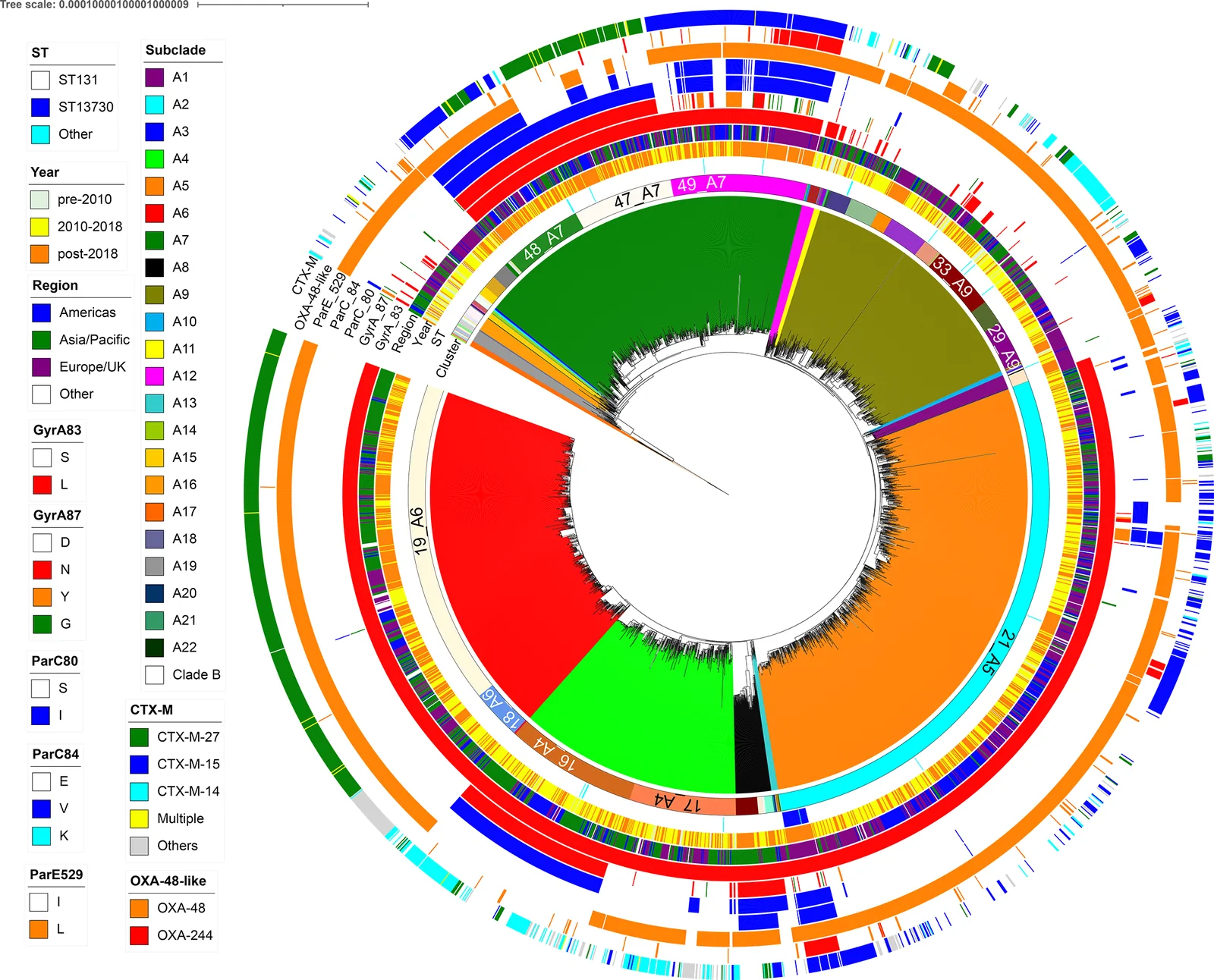
A core SNP–based phylogenetic tree of 2,905 *E. coli* ST131 clade A genomes. The maximum-likelihood phylogram was constructed based on a total of 40,705 core SNPs. The tree additionally includes the clade B reference genome SaT040, which was used as the root. The outgroup tip was omitted from the displayed tree to improve visualization of the ST131-A phylogeny and outer annotation rings. Subclades are indicated by coloured shading within the phylogram, and STs are shown outside the cluster ring. Frequent clusters are labelled within the cluster annotation strip using the cluster number followed by the corresponding subclade designation (e.g. 16_A4). The complete rooted phylogeny, including SaT040 and a collapsed representation showing the branching structure and maximum-likelihood support of the ST131-A groups are provided in Fig. S1.

The ST131-A subclades were further divided into 79 clusters at the second BAPS level (Figs 1 and S1). Hereafter, clusters are denoted by the cluster number followed by the subclade designation (e.g. 16_A4). The ten most frequent clusters were 21_A5 (29%), 19_A6 (17%), 49_A7 (7%), 16_A4 (7%), 17_A4 (6%), 47_A7 (5%), 48_A7 (4%), 33_A9 (3%), 18_A6 (3%) and 29_A9 (2%) (Tables 1 and S2). These frequent clusters represented 83% of the total ST131-A population.

To assess the robustness of the BAPS population structure, we performed bootstrap co-clustering analysis (Fig. S2). When the five dominant subclades were treated as single homogeneous groups, their mean within-subclade bootstrap values were relatively low (<80; Table S2). However, all clusters within A4, A6, A7 and A9 showed high within-cluster bootstrap support (>90), except for cluster 35_A9, which showed moderate support (mean bootstrap value, 81). In subclade A5, BAPS level 2 did not further subdivide the subclade, although the bootstrap co-clustering heatmap showed two blocks with high within-block co-clustering (Fig. S2). The remaining smaller subclades showed high mean within-subclade bootstrap values (>94) and were more consistently recovered as discrete clusters. Sensitivity analysis using three more conservative prior settings assigned all ST131-A genomes to a single cluster and did not provide additional resolution of the observed population structure.

All subclades and clusters were monophyletic in the maximum-likelihood tree (Fig. 1 and S1). Among the five dominant subclades, the most recent common ancestor (MRCA) nodes defining A6 and A9 met both branch-support criteria, A5 met one of the two criteria and A4 and A7 met neither criterion (Table S2). Among the frequent clusters, 18_A6, 19_A6, 47_A7, 48_A7 and 29_A9 met both criteria; 16_A4 and 21_A5 met one of the two criteria; and 17_A4, 49_A7 and 33_A9 met neither criterion. Among the remaining non-singleton groups, 12 of 14 subclades (86%) and 34 of 52 clusters (65%) met both criteria.

Overall, most ST131-A genomes contained *fimH*41 (92%) and were positive for serotypes O16 (93%) and H5 (96%) [Table 1]. The proportional representation of subclades A4 and A9 declined from the pre-2010 to the post-2018 period, whereas A5, A6 and A7 increased over time (Fig. 2a). The dominant subclades showed distinct geographic distributions (Fig. 2b and Table 1). A4 and A6 were associated with the Asia/Pacific region, A5 with the Americas and Europe/UK, A7 with the Americas and A9 with Europe/UK (*P*<0.05).

**Fig. 2.**
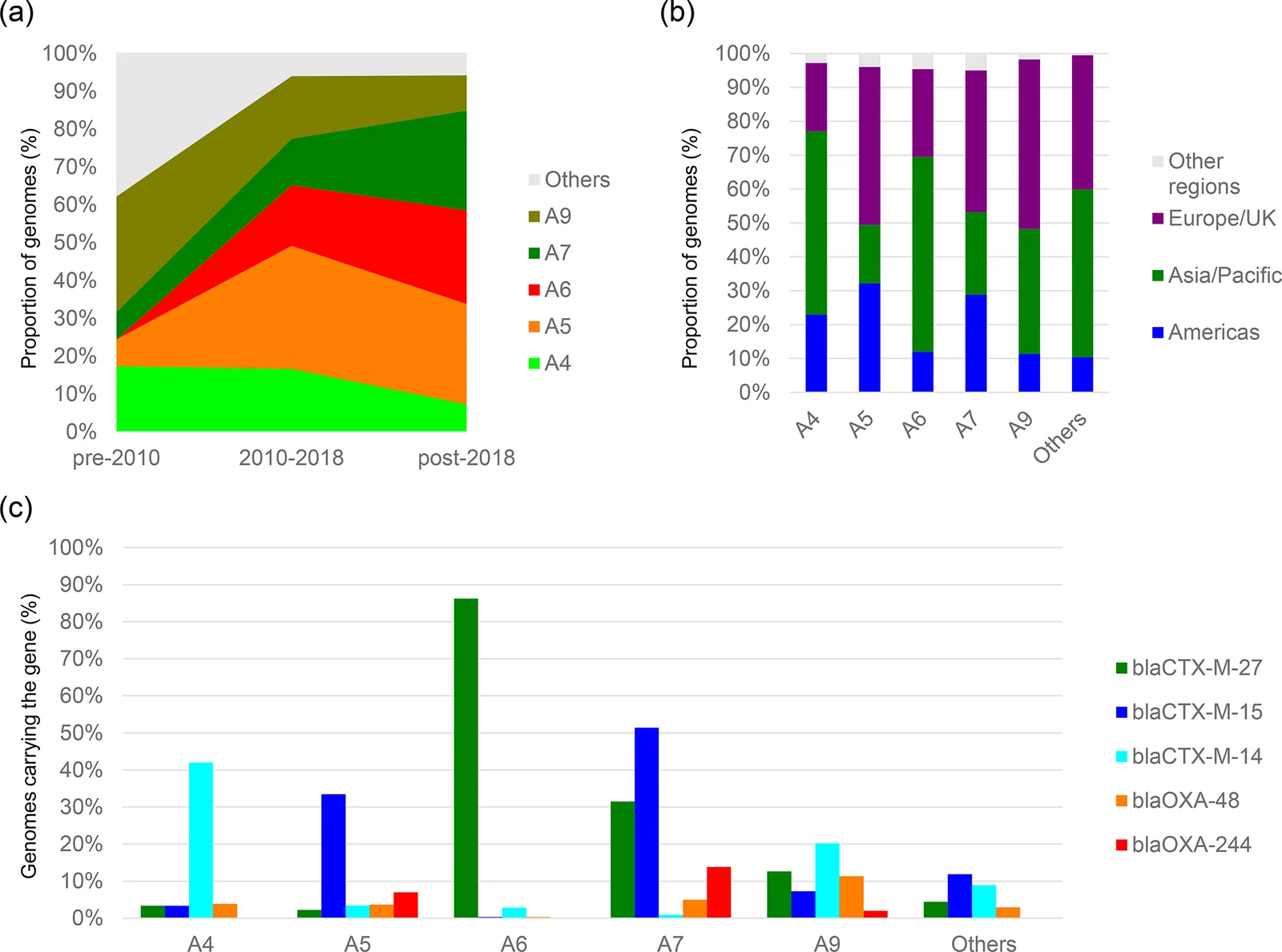
Temporal and geographic distributions of ST131-A subclades and carriage of major CTX-M and OXA-48-like genes. (**a**) Proportional distribution of the five dominant subclades (**A4, A5, A6, A7 and A9**) and other subclades across three collection periods. Colours are harmonized with those used for subclades in Fig. 1. (**b**) Proportional geographic composition of genomes within each subclade group. (**c**) Proportions of genomes carrying major CTX-M and OXA-48-like genes within each subclade group. All gene–subclade associations shown were statistically significant, except those between OXA-48 and subclades A4, A5 and A7 (each subclade versus all other ST131-A genomes; Table 2). ‘Others’ denotes all subclades other than A4, A5, A6, A7 and A9.

### Dominant ST131-A subclades were linked with specific AMR determinants and VAFs

The most common QRDR mutations in the overall ST131-A population were *gyrA* S83L (82%) and *parE* I529L (86%; Table 2). Additional mutations, including *gyrA* D87N, *parC* S80I and *parC* E84V, were less frequent overall but showed marked subclade-specific distributions. *gyrA* D87N and *parC* S80I were particularly frequent in A4 and A7, and *parC* E84V was predominantly associated with A7 (*P*<0.05). In contrast, *parE* L445H and *parE* S458A were almost restricted to A4 (Table 2).

**Table 2.** AMR genes and plasmids of dominant subclades among ST131-A

	*A4 *n*=357 **(12%)**	A5 *n*=840 **(29%)**	A6 *n*=567 **(20%)**	A7 *n*=543 **(19%)**	A9 *n*=396 **(14%)**	All *n*=2,905 **(100%)**
QRDR mutations:						
*gyrA* S83L	357 (100%)†	840 (100%)†	567 (100%)†	474 (87%)†	46 (12%)†	2,390 (82%)
*gyrA* D87N	204 (57%)†	10 (1%)†	0†	292 (54%)†	7 (2%)†	574 (20%)
*parC* S80I	213 (60%)†	101 (12%)†	1 (<1%)†	421 (78%)†	4 (1%)†	802 (28%)
*parC* E84V	0†	57 (7%)†	0†	300 (55%)†	1 (<1%)†	404 (14%)
*parE* I529L	145 (41%)†	796 (95%)†	567 (100%)†	420 (77%)†	388 (98%)†	2,504 (86%)
*parE* L445H	196 (55%)†	0†	0†	0†	0†	209 (7%)
*parE* S458A	140 (39%)†	6 (<1%)†	0†	0†	0†	146 (5%)
β-lactamases						
CMYs	8 (2%)	19 (2%)	10 (2%)	13 (2%)	8 (2%)	85 (3%)
DHA-1	0	1 (<1%)†	0†	5 (<1%)	14 (4%)†	24 (<1%)
CTX-M-14	150 (42%)†	29 (3%)†	16 (3%)†	5 (<1%)†	80 (20%)†	298 (10%)
CTX-M-15	12 (3%)†	281 (33%)†	2 (<1%)†	279 (51%)†	29 (7%)†	627 (22%)
CTX-M-27	12 (3%)†	19 (2%)†	489 (86%)†	171 (31%)†	50 (13%)†	750 (26%)
CTX-M others	43 (12%)†	42 (5%)	57 (10%)†	9 (2%)†	18 (5%)	180 (6%)
KPCs	10 (3%)	20 (2%)	18 (3%)	12 (2%)	2 (<1%)†	68 (2%)
NDMs	3 (<1%)	6 (<1%)	0†	18 (3%)†	0	29 (<1%)
IMPs	4 (1%)	0	0	0	8 (2%)†	14 (<1%)
VIMs	0	1 (<1%)	0	12 (2%)†	0	13 (<1%)
OXA-48	14 (4%)	31 (4%)	2 (<1%)†	27 (5%)	45 (11%)†	125 (4%)
OXA-244	0†	59 (7%)†	0†	75 (14%)†	8 (2%)†	142 (5%)
OXA others	4 (1%)	18 (2%)	1 (<1%)†	15 (3%)	3 (<1%)	52 (2%)
SHVs	0	2 (<1%)	0	13 (2%)†	0	18 (<1%)
TEM-1	234 (66%)	649 (77%)†	535 (94%)†	158 (29%)†	222 (56%)†	1,900 (65%)
TEM others	59 (17%)†	31 (4%)	11 (2%)†	11 (2%)†	9 (2%)	131 (5%)
Aminoglycoside modifying enzymes (>10% prevalence):						
*aac(3)-IId*	175 (49%)†	272 (32%)	398 (70%)†	88 (16%)†	58 (15%)†	1,015 (35%)
*aadA5*	163 (46%)†	446 (53%)	429 (76%)†	359 (66%)†	129 (33%)†	1,554 (53%)
*aph(3’’)-Ib*	187 (52%)	399 (48%)†	426 (75%)†	254 (47%)	192 (48%)	1,504 (52%)
*aph(6)-Id*	184 (52%)	388 (46%)†	424 (75%)†	255 (47%)	194 (49%)	1,491 (51%)
Other AMR determinants (>10% prevalence):						
*dfrA14*	8 (2%)†	53 (6%)	2 (<1%)†	28 (5%)	54 (14%)†	158 (5%)
*dfrA17*	159 (45%)†	454 (54%)	428 (75%)†	363 (67%)†	170 (43%)†	1,602 (55%)
*erm(B)*	7 (2%)†	27 (3%)†	1 (<1%)†	106 (20%)†	7 (2%)†	153 (5%)
*mph(A)*	165 (46%)†	482 (57%)	431 (76%)†	379 (70%)†	196 (49%)†	1,708 (59%)
*sul1*	174 (49%)†	447 (53%)	428 (75%)†	378 (70%)†	150 (38%)†	1,631 (56%)
*sul2*	190 (53%)	392 (47%)†	427 (75%)†	264 (49%)	193 (49%)	1,509 (52%)
*tet(A)*	157 (44%)	377 (45%)	424 (75%)†	246 (45%)	99 (25%)†	1,338 (46%)
*tet(B)*	19 (5%)†	53 (6%)†	0†	35 (6%)	104 (26%)†	261 (9%)
Plasmids (>10% prevalence)						
Col156	209 (59%)†	687 (82%)†	509 (90%)†	425 (78%)	256 (65%)†	2,193 (75%)
IncI1-I(Alpha)	50 (14%)	122 (15%)†	9 (2%)†	50 (9%)	42 (11%)	292 (10%)
FAB profiles (>10% prevalence)‡						
F29:A-:B10	4 (1%)†	511 (61%)†	493 (87%)†	21 (4%)†	138 (35%)†	1,209 (42%)
F1:A2:B20	225 (63%)†	6 (<1%)†	0†	203 (37%)†	49 (12%)	486 (17%)
F1:A1:B16	0†	0†	0†	124 (23%)†	0†	124 (4%)
F1:A1:B1	0†	1 (<1%)†	0†	1 (<1%)†	49 (12%)†	63 (2%)
F2:A-:B10	1 (<1%)†	81 (10%)†	20 (4%)	3 (<1%)†	26 (7%)	143 (5%)
F2:A2:B20	40 (11%)†	0†	0†	16 (3%)	1 (<1%)†	58 (2%)
Any FAB assigned	348 (97%)	830 (99%)†	560 (99%)†	516 (95%)	363 (92%)†	2,794 (96%)

*ST131-A subclades were defined at the first BAPS level clustering.

†Bonferroni-corrected *P*<0.05 by Fisher’s exact test, comparing each subclade with all remaining ST131-A genomes.

‡Individual FAB profiles with a prevalence of >10% in at least one subclade are shown separately. ‘Any FAB assigned’ includes all assigned FAB profiles, including those not shown individually.

The three major CTX-M variants were *bla*_CTX-M-27_ (26%), *bla*_CTX-M-15_ (22%) and *bla*_CTX-M-14_ (10%) (Table 2). *bla*_CTX-M-27_ was significantly associated with A6 (86%) and A7 (31%), *bla*_CTX-M-15_ with A5 (33%) and A7 (51%) and *bla*_CTX-M-14_ with A4 (42%) and A9 (20%; Table 2 and Fig. 2c; *P*<0.05). Carbapenemase genes were uncommon overall, with *bla*_OXA-244_ (5%) and *bla*_OXA-48_ (4%) being the most frequent carbapenemases. *bla*_OXA-244_ was significantly associated with A5 (7%) and A7 (14%), whereas *bla*_OXA-48_ was significantly associated with A9 (11%) (Fig. 2c and Table 2). Other acquired AMR determinants were also common and showed subclade-specific distributions (Table 2). For example, *bla*_TEM-1_, *aadA5*, *aph(3’’)-Ib, aph(6)-Id*, *dfrA17*, *mph(A)*, *sul1* and *sul2* were each present in more than half of ST131-A genomes, and several of these determinants were significantly associated with A6.

Certain *E. coli* VAFs were frequent (>60%) among all ST131-A subclades, namely *papA-F, iha, fimH, sat, fyuA, iutA, kpsMII_K5, usp, traT and ompT* (Table S3). Specific VAFs were linked to certain subclades: *kpsM_K15* and *ompT* with ST131-A4, *papA_F43*, *hra, astA, ireA, kpsMII_K5, traT* and *ompT* with ST131-A5, *iha*, *hlyA*, *sat*, *astA*, *cnf1*, *iutA*, *kpsMII_K5, traT* and *ompT* with ST131-A6, *papA_F43*, *iha*, *sat*, *iutA* and *kpsM II* with ST131-A7, *papA_F43*, *iha*, *afaA*, *iutA* and *kpsMII_K5* with ST131-A9 (Table S3).

### Major CTX-M and OXA-48-like genes were associated with certain clusters within ST131-A subclades

Major CTX-M and OXA-48-like genes showed cluster-specific associations within ST131-A (Fig. 3a). The complete distributions of these genes across ST131-A subclades and clusters are shown in Table 3, whereas cluster-specific statistical comparisons are provided in Table S4. *bla*_CTX-M-27_ was detected in 750/2,905 (26%) ST131-A genomes; of these, 486 (65%) belonged to 19_A6 and 140 (19%) to 47_A7. Within these clusters, *bla*_CTX-M-27_ was present in 486/489 (99%) and 140/150 (93%) genomes, respectively and was significantly associated with both clusters compared with all remaining ST131-A genomes (*P*<0.05 for each; Fig. 3a and Table S4). Both *bla*_CTX-M-27_-associated clusters showed global distributions. Cluster 47_A7 was frequently identified in the USA and Australia (Fig. 3b).

**Fig. 3.**
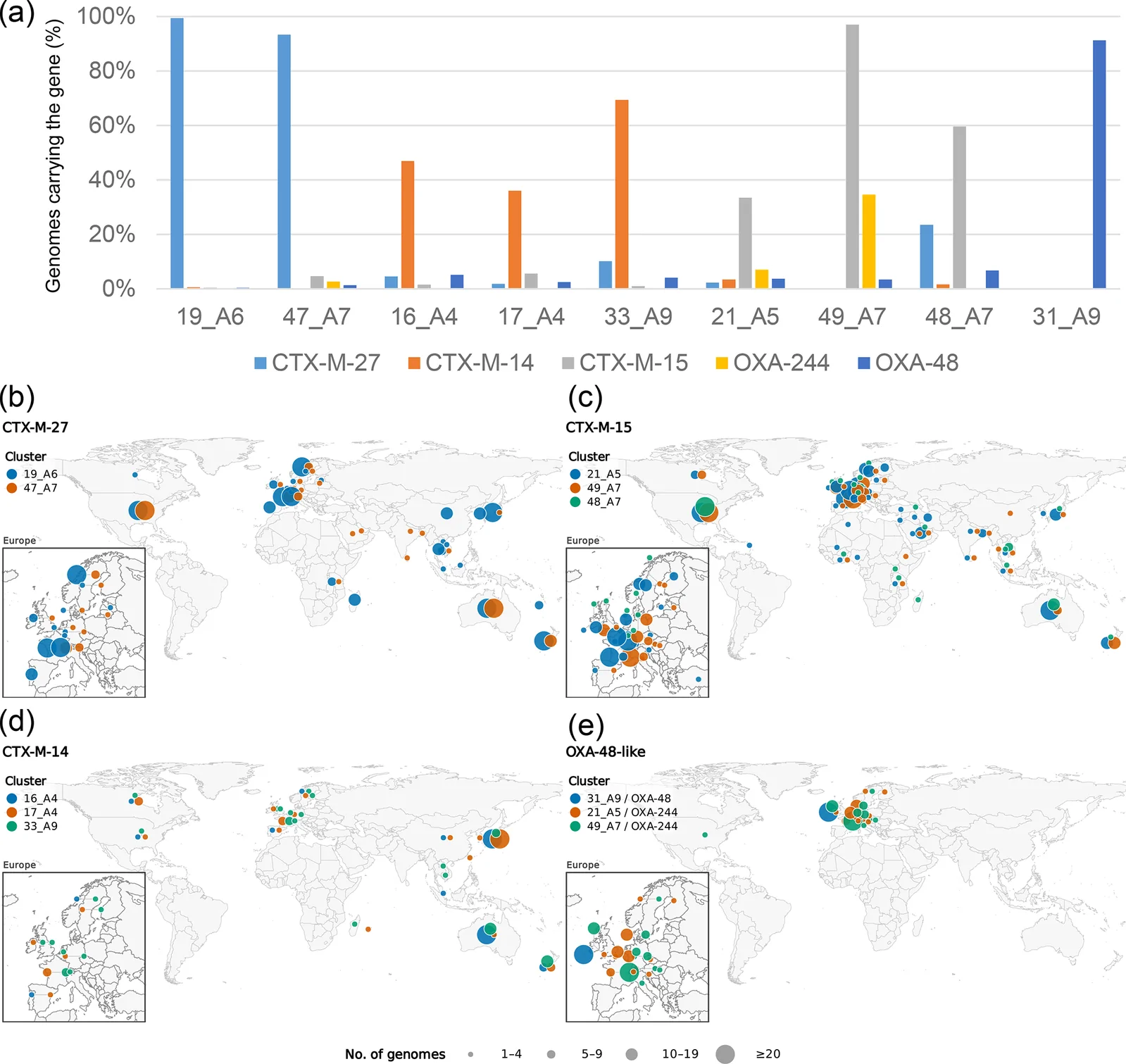
Distribution of major CTX-M and OXA-48-like genes in associated ST131-A clusters. (**a**) Proportions of genomes carrying major CTX-M and OXA-48-like β-lactamase genes within the indicated ST131-A clusters. Statistical comparisons of each cluster with all remaining ST131-A genomes are provided in Table S4. (**b–e**) Geographic distribution of gene-carrying genomes within the indicated clusters. Circle colours indicate clusters, and circle sizes represent the number of genomes identified in each country. Insets show enlarged views of Europe.

**Table 3.** Distribution of major CTX-M and OXA-48-like genes across ST131-A subclades and clusters

β-lactamase gene (no. of genome)	No. of subclade	ST131-A subclade, n (% of gene-positive genome)	ST131-A cluster, n (% of gene-positive genome)
CTX-M-27	7	A2 *n*=3 (<1%)	5_A2 *n*=3 (<1%)
*n*=750/2,905 (26%)		A4 *n*=12 (2%)	16_A4 *n*=9 (1%), 13_A4 *n*=3 (<1%)
		A5 *n*=19 (3%)	21_A5 *n*=19 (3%)
		**A6 *n*=489 (65%)**	18_A6 *n*=3 (<1%), **19_A6 *n*=486 (65%)**
		**A7 *n*=171 (23%)**	**47_A7 *n*=140 (19%),** 48_A7 *n*=28 (4%), 52_A7 *n*=2 (<1%), 53_A7 *n*=1 (<1%)
		A8 *n*=6 (<1%)	3_A8 *n*=6 (<1%)
		A9 *n*=50 (7%)	29_A9 *n*=2 (<1%), 30_A9 *n*=7 (<1%), 32_A9 *n*=4 (<1%), 33_A9 *n*=10 (1%), 35_A9 *n*=24 (3%), 36_A9 *n*=3 (<1%)
CTX-M-15	11	A1 *n*=10 (2%)	7_A1 *n*=10 (2%)
*n*=627/2,905 (22%)		A4 *n*=12 (2%)	16_A4 *n*=3 (<1%), 17_A4 *n*=9 (1%)
		**A5 *n*=281 (45%)**	**21_A5 *n*=281 (45%)**
		A6 *n*=2 (<1%)	19_A6 *n*=2 (<1%)
		**A7 *n*=279 (44%)**	47_A7 *n*=7 (1%), **48_A7 *n*=71 (11%)**, **49_A7 *n*=199 (32%),** 52_A7 *n*=1 (<1%), 54_A7 *n*=1 (<1%)
		A8 *n*=10 (2%)	2_A8 *n*=9 (1%), 4_A8 *n*=1 (<1%)
		A9 *n*=29 (5%)	29_A9 *n*=20 (3%), 32_A9 *n*=2 (<1%), 33_A9 *n*=1 (<1%), 34_A9 *n*=3 (<1%), 35_A9 *n*=1 (<1%), 36_A9 *n*=2 (<1%)
		A12 *n*=1 (<1%)	46_A12 *n*=1 (<1%)
		A14 *n*=1 (<1%)	58_A14 *n*=1 (<1%)
		A16 *n*=1 (<1%)	65_A16 *n*=1 (<1%)
		A19 *n*=1 (<1%)	75_A19 *n*=1 (<1%)
CTX-M-14	11	**A4 *n*=150 (50%)**	**16_A4 *n*=92 (31%), 17_A4 *n*=58 (19%)**
*n*=298/2,905 (10%)		A5 *n*=29 (10%)	21_A5 *n*=29 (10%)
		A6 *n*=16 (5%)	18_A6 *n*=11 (4%), 19_A6 *n*=3 (1%), 20_A6 *n*=2 (1%)
		A7 *n*=5 (2%)	48_A7 *n*=2 (1%), 52_A7 *n*=1 (<1%), 53_A7 *n*=2 (1%)
		A8 *n*=3 (1%)	1_A8 *n*=1 (<1%), 3_A8 *n*=2 (1%)
		**A9 *n*=80 (27%)**	30_A9 *n*=2 (1%), **33_A9 *n*=68 (23%),** 34_A9 *n*=3 (1%), 35_A9 *n*=2 (1%), 36_A9 *n*=4 (1%), 38_A9 *n*=1 (<1%)
		A11 *n*=6 (2%)	40_A11 *n*=6 (2%)
		A12 *n*=2 (<1%)	46_A12 *n*=2 (<1%)
		A17 *n*=3 (1%)	66_A17 *n*=3 (1%)
		A19 *n*=3 (1%)	73_A19 *n*=3 (1%)
		A20 *n*=1 (<1%)	77_A20 *n*=1 (<1%)
OXA-48*	9	A1 *n*=1 (<1%)	9_A1 *n*=1 (<1%)
*n*=125/2,905 (4%)		A3 *n*=2 (2%)	6_A3 *n*=2 (2%)
		A4 *n*=14 (11%)	16_A4 *n*=10 (8%), 17_A4 *n*=4 (3%)
		A5 *n*=31 (25%)	21_A5 *n*=31 (25%)
		A6 *n*=2 (2%)	19_A6 *n*=2 (2%)
		A7 *n*=27 (22%)	47_A7 *n*=2 (2%), 48_A7 *n*=8 (6%), 49_A7 *n*=7 (6%), 54_A7 *n*=8 (6%), 55_A7 *n*=2 (2%)
		**A9 *n*=45 (36%)**	28_A9 *n*=2 (2%), 29_A9 *n*=7 (6%), 30_A9 *n*=5 (4%), **31_A9 *n*=21 (17%),** 32_A9 *n*=4 (3%), 33_A9 *n*=4 (3%), 35_A9 *n*=1 (<1%), 36_A9 *n*=1 (<1%)
		A13 *n*=2 (2%)	22_A13 *n*=2 (2%)
		A15 *n*=1 (<1%)	60_A15 *n*=1 (<1%)
OXA-244	3	**A5 *n*=59 (42%)**	**21_A5 *n*=59 (42%)**
*n*=142/2,905 (5%)		**A7 *n*=75 (53%)**	47_A7 *n*=4 (3%), **49_A7 *n*=71 (50%)**
		A9 *n*=8 (6%)	29_A9 *n*=8 (6%)

Dominant subclades and frequent clusters are highlighted.

*Among OXA-48 gene-positive genomes, pOXA-48a-like plasmid backbone sequences showing >90% reference coverage and nucleotide identity were detected in 100/125 (80%) genomes: A1, 1/1 (100%); A3, 2/2 (100%); A4, 11/14 (79%); A5, 26/31 (84%); A6, 2/2 (100%); A7, 12/27 (44%); A9, 43/45 (96%); A13, 2/2 (100%); and A15, 1/1 (100%).

*bla*_CTX-M-15_ was detected in 627/2,905 (22%) ST131-A genomes; of these, 281 (45%) belonged to 21_A5, 199 (32%) to 49_A7 and 71 (11%) to 48_A7. Within these clusters, *bla*_CTX-M-15_ was present in 281/840 (33%), 199/205 (97%) and 71/119 (60%) genomes, respectively, and was significantly associated with all three clusters compared with all remaining ST131-A genomes (*P*<0.05 for each; Fig. 3a and Table S4). These three clusters showed broad global distributions (Fig. 3c).

*bla*_CTX-M-14_ was detected in 298/2,905 (10%) ST131-A genomes; of these, 92 (31%) belonged to 16_A4, 58 (19%) to 17_A4 and 68 (23%) to 33_A9. Within these clusters, *bla*_CTX-M-14_ was present in 92/196 (47%), 58/161 (36%) and 68/98 (69%) genomes, respectively, and was significantly associated with all three clusters compared with all remaining ST131-A genomes (*P*<0.05 for each; Fig. 3a and Table S4). The three *bla*_CTX-M-14_-associated clusters showed broad geographic distributions, particularly in Japan, Australia and New Zealand (Fig. 3d).

*bla*_OXA-48_ was detected in 125/2,905 (4%) ST131-A genomes; of these, 21 (17%) belonged to 31_A9. Within 31_A9, *bla*_OXA-48_ was present in 21/23 (91%) genomes and was significantly associated with this cluster compared with all remaining ST131-A genomes (*P*<0.05; Fig. 3a and Table S4). The *bla*_OXA-48_-positive 31_A9 genomes were predominantly identified in Ireland during 2021–23 (Fig. 3e). pOXA-48a-like plasmid backbone sequences showing >90% reference coverage and nucleotide identity were detected in 100/125 (80%) *bla*_OXA-48_-positive genomes, compared with 8/2,780 (0.3%) *bla*_OXA-48_-negative genomes (*P*<0.001). These sequences were detected among *bla*_OXA-48_-positive genomes across all nine ST131-A subclades and in all *bla*_OXA-48_-positive 31_A9 genomes (Table 3).

*bla*_OXA-244_ was detected in 142/2,905 (5%) ST131-A genomes; of these, 59 (42%) belonged to 21_A5 and 71 (50%) to 49_A7. Within these clusters, *bla*_OXA-244_ was present in 59/840 (7%) and 71/205 (35%) genomes, respectively, and was significantly associated with both clusters compared with all remaining ST131-A genomes (*P*<0.05 for each; Fig. 3a and Table S4). Both *bla*_OXA-244_-associated clusters were concentrated in Europe but showed different country-level distributions (Fig. 3e). Among the 71 *bla*_OXA-244_-positive 49_A7 genomes, 70 also carried *bla*_CTX-M-15_.

### The dominant ST131-A subclades diversified mainly during the 1990s

Root-to-tip regression analysis showed a modest but positive temporal signal in the ST131-A dataset (R^²^=0.16; slope=5.94×10^−7^ substitutions/site/year; Fig. S3). In the BEAST analysis, path-sampling marginal likelihood estimation supported the strict clock model over the optimized relaxed clock model (Table S5), and the strict clock was, therefore, used for all reported time-scaled phylogenetic estimates. The three independent BEAST runs showed stable posterior traces and effective sample sizes exceeded 200 for all parameters (Table S6). Under the strict clock model, the estimated mean substitution rate of ST131-A is 5.58×10^−7^ substitutions per site per year (95% highest posterior density interval [HPD], 5.39×10^−7^–5.76×10^−7^), and the MRCA of ST131-A was estimated to date to 1938 (HPD, 1931–1944; Fig. 4). MRCA estimates with 95% HPD intervals and node posterior probabilities are shown in Fig. S4 and summarized in Table S2. The time-scaled phylogeny placed A17 among the earliest-diverging ST131-A subclades, followed by the divergence of several other basal lineages before 1990 and the diversification of the five dominant subclades mainly during the 1990s. A4, A5, A7 and A9 were estimated to diverge during the early 1990s, with substantially overlapping 95% HPD intervals. In contrast, A6 was estimated to diverge later, in the late 1990s, with a 95% HPD interval that did not overlap those of the other four dominant subclades. The MRCAs of frequent clusters were estimated to date to the 1990s, whereas those of 18_A6, 19_A6 and 47_A7 were estimated to date to the 2000s. The MRCA nodes of A4, A5, A6 and A9 had posterior probabilities of 0.997–1, whereas that of A7 had a lower posterior probability of 0.69. Similarly, the MRCA nodes of the frequent clusters had posterior probabilities of 0.997–1, except for 33_A9, which had a posterior probability of 0.238. In the time-scaled phylogeny, eight of the ten frequent clusters were monophyletic, whereas 17_A4 and 49_A7 were each represented by a small number of locally coherent components rather than being broadly intermixed with other clusters (Fig. S4 and Table S2).

**Fig. 4.**
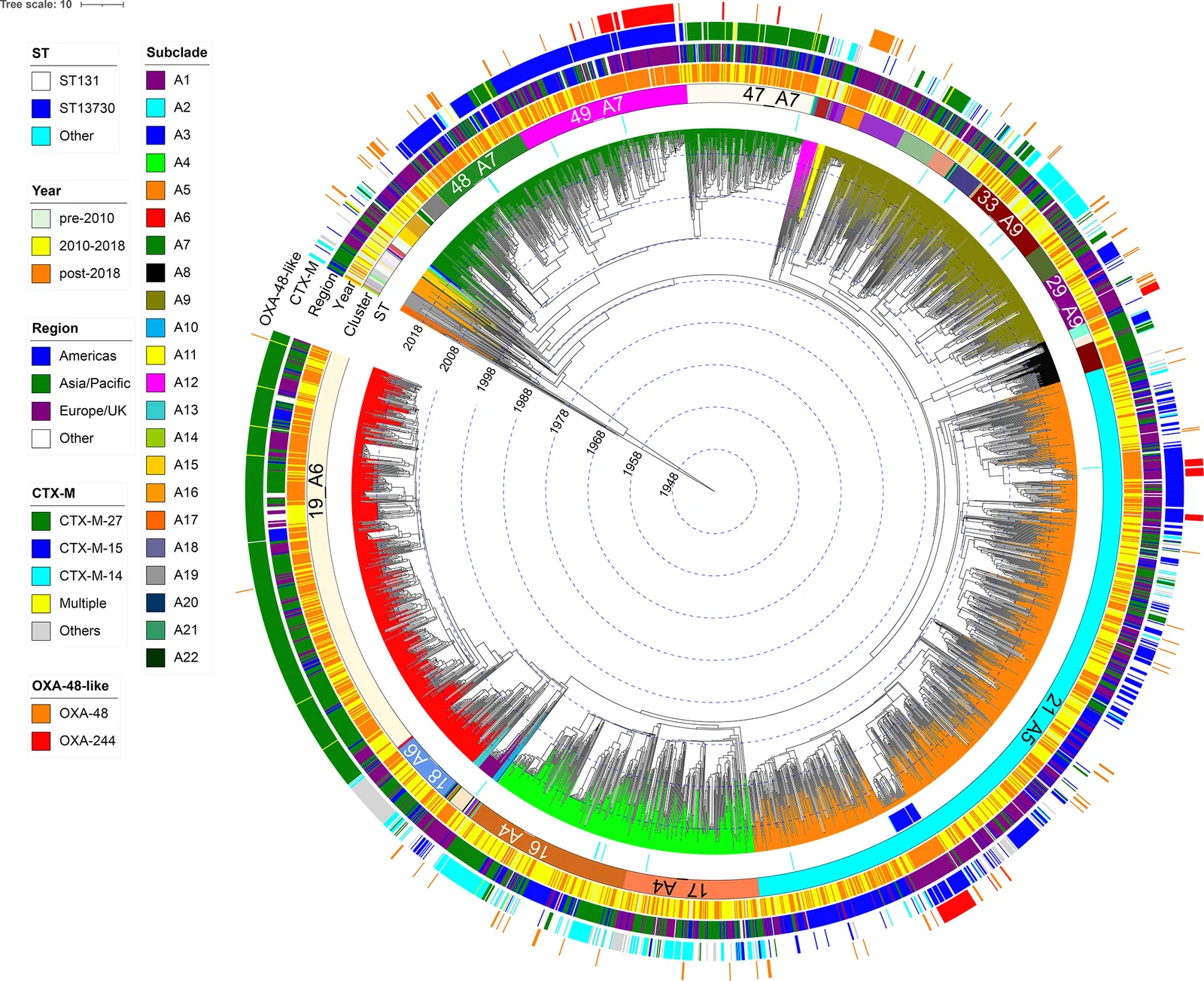
A time-scaled phylogeny of 2,905 *E. coli* ST131 clade A genomes. Phylogenetic reconstruction was performed using a general time-reversible substitution, a strict molecular clock and a Bayesian skyline population model. The clade B reference genome SaT040 was included in the BEAST analysis but omitted from this figure because its deep divergence extended the timescale and obscured the internal diversification within clade A. Frequent clusters are labelled within the cluster annotation strip using the cluster number followed by the corresponding subclade designation (e.g. 16_A4).

### Temporal diversification of clusters associated with major CTX-M and OXA-48-like genes

The seven frequent clusters associated with CTX-M-27, CTX-M-15 or CTX-M-14 had estimated MRCA dates ranging from 1992 to 2005 (Fig. 4 and Table S2). The CTX-M-14-associated clusters 16_A4, 17_A4 and 33_A9 and the CTX-M-15-associated clusters 21_A5 and 49_A7 were estimated to have diverged during the 1990s. In contrast, the CTX-M-27-associated clusters 19_A6 and 47_A7 were estimated to diverge later, during the 2000s. The *bla*_OXA-244_-positive genomes were identified within two CTX-M-15-associated clusters, 21_A5 and 49_A7. The earliest *bla*_OXA-244_-positive isolates in these clusters in our dataset were identified much later than the estimated MRCAs of these clusters, in 2021 and 2019, respectively. The OXA-48-associated cluster 31_A9 had an estimated MRCA dating to 1998, whereas the earliest *bla*_OXA-48_-positive isolate in this cluster was identified in 2021 (Fig. 4 and Table S2).

### Complete genome analysis identified the locations and mobile genetic elements associated with CTX-M and OXA-48-like genes

A total of 178 complete genomes included the following 14 subclades, predominantly A5 (*n*=44), A6 (*n*=38), A7 (*n*=34), A9 (*n*=26) and A4 (*n*=17); the remaining 19 genomes represented nine other subclades. These genomes carried an average of 1.7 plasmids (range, 0–7), consisting of an average of 1.1 IncF F:A:B formula (FAB)-typeable plasmids (range, 0–3), 0.3 non-IncF plasmids (range, 0–5) and 0.4 small circular plasmids <10 kb in size (range, 0–2). Pling analysis of 262 non-small circular plasmids identified 11 multi-plasmid communities (communities 0–10), 15 singleton communities and three unassigned hub plasmids (Table S7). Pling communities represent broad groups based on shared sequence content and correspond to plasmid replicon types in our dataset (Table S7). Community_0 was the largest, comprising 196 predominantly IncF plasmids divided into 44 subcommunities and encompassing multiple IncF FAB profiles and host clusters. Within each community, subcommunities further group structurally related plasmids, reflecting differences in rearrangements and sequence gain or loss. In community_0, these subcommunities showed partial correspondence with FAB profiles; the largest, subcommunity_45, comprised 108 plasmids and was dominated by the F29:A-:B10 and related profiles (81%) (Table S8 and Fig. S5a). Several additional communities or subcommunities were associated with plasmids carrying CTX-M or carbapenemase genes, as described below.

Among 53 complete genomes that contained *bla*_CTX-M-27_, 36 of the genes were found on the chromosome of cluster 19_A6. These genes were located within IS*Ecp1-bla*_CTX-M-27_**-**ΔIS*903D* elements inserted into the *gspD* gene that generated 5 bp direct repeats (Fig. 3 and Table S9). A single genome from cluster 52_A7 also contained chromosomal *bla*_CTX-M-27_, which was situated within IS*Ecp1-bla*_CTX-M-27_**-**ΔIS*903D-iroN*-ΔTn2 element that was inserted within a different chromosomal gene. The CTX-M-27 genes were present on plasmids within the remaining 16 genomes. In the Pling analysis, six F1:A2:B20 plasmids from 47_A7, two from 48_A7 and one F1-like:A2:B20 plasmid from 21_A5 grouped together in subcommunity_43 (Table S8 and Fig. S5b). The F1:A2:B20 plasmid of the ST131-C1-M27 strain Ecol_732 was also assigned to this subcommunity and showed >99% identity and 82–100% coverage with the plasmids in this subcommunity. The remaining seven plasmids, comprising F2:A-:B10 (*n*=2) and one each of F1:A1:B20, F2:A2:B20, F29:A-:B10, F35:A2:B20 and F59-like:A-:B-, were distributed across five other subcommunities. In 15/16 of these plasmids, the CTX-M-27 genes were incorporated into ΔIS*Ecp1-bla*_CTX-M-27_**-**ΔIS*903D* elements that were flanked by IS*26* and differed from those on the chromosomes. One genome in ST131-A9 carried an *eitABCD*-IS*Ecp1-bla*_CTX-M-27_**-**ΔIS*903D***-***malB* structure, which represented a *bla*_CTX-M-14_**-**like element.

Of 28 genomes that contained *bla*_CTX-M-15_, half were situated within the chromosomes of ST131-A7 (49_A7 [*n*=8]; 48_A7 [*n*=1]) and ST131-A5 (21_A5 [*n*=5]). The genes were located within IS*Ecp1-bla*_CTX-M-15_**-***orf477* elements, with or without ΔTn2, inserted at several chromosomal sites that differed between subclades (Fig. 5 and Table S10). The remaining fourteen CTX-M-15 genes were present on plasmids (Fig. 5 and Table S10). The IncFIB(H89-PhagePlasmid) was the most common replicon type, detected in clusters 21_A5 (*n*=4) and 48_A7 (*n*=1). These IncFIB(H89-PhagePlasmid) plasmids grouped within subcommunities 1 and 2 of Pling community_1 (Table S8 and Fig. S5c). The reference IncFIB(H89-PhagePlasmid) plasmid pECOH89 was also assigned to this subcommunity and showed >98% identity and 91–100% coverage with the plasmids in this subcommunity. Four IncF plasmids (F1:A1:B66, F2:A-:B-, F2:A2:B20 and F22,36:A1:B1) were distributed among clusters 16_A4, 21_A5 and 48_A7. IncI (*n*=4) and IncB/O/K/Z (*n*=1) plasmids carrying *bla*_CTX-M-15_ were restricted to cluster 21_A5. These plasmids carried either IS*Ecp1-bla*_CTX-M-15_**-***orf477* or IS*26*-ΔIS*Ecp1-bla*_CTX-M-15_**-***orf477*, with or without ΔTn2 elements.

**Fig. 5.**
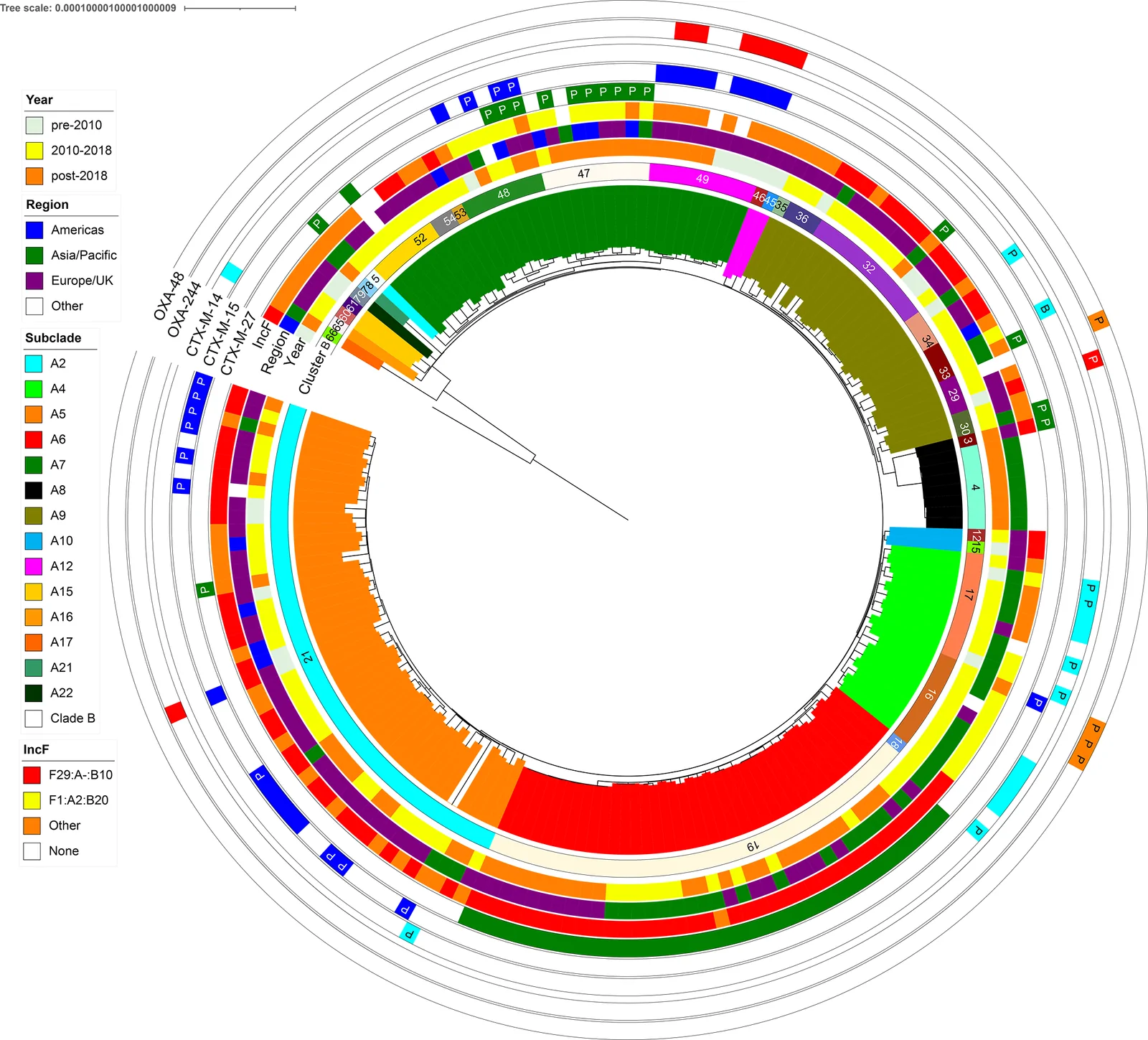
A core SNP–based phylogenetic tree of 178 *E. coli* ST131 clade A genomes. The maximum-likelihood phylogram was constructed based on a total of 5,117 core SNPs. The tree additionally includes the clade B reference genome SaT040, which was used as the root. Subclades are indicated by coloured shading within the phylogram. Within the major CTX-M and carbapenemase gene tracks, ‘P’ indicates that the corresponding gene was located on a plasmid; coloured cells without ‘P’ indicate chromosomal location. Blank cells indicate absence of the corresponding gene.

Of 15 genomes that contained *bla*_CTX-M-14_, seven harboured the gene on the chromosome (ST131-A4 [cluster 16, *n*=4; cluster 17, *n*=2] and ST131-A7 [*n*=1]), while seven genes were present on plasmids, and one genome contained both chromosomal and plasmid genes (Fig. 5 and Table S11). Within the chromosome, the gene was located within IS*Ecp1-bla*_CTX-M-14_**-**ΔIS*903D* (with or without *malB*) or IS*Ecp1-bla*_CTX-M-14_**-**IS*903D-iroN* elements and was inserted at various chromosomal sites. Three of the ST131-A4 genomes contained multiple copies of *bla*_CTX-M-14_ inserted at the same chromosomal site. Among plasmid-encoded genomes, IncFII was the most common replicon type (F2:A-:B-, *n*=3; F35:A-:B-, *n*=1), which carried the *eitABCD*-IS*Ecp1-bla*_CTX-M-14_**-**ΔIS*903D***-***malB* structure. Of these, three plasmids grouped in subcommunity_40 together with one *bla*_CTX-M-27_-carrying plasmid (Table S11 and Fig. S5d). The F2:A-:B- plasmid of the clade C1-non-M27 strain TUM2802 was also assigned to this subcommunity and showed >99% identity and 42–96% coverage with the plasmids in this subcommunity. IncB/O/K/Z plasmids carrying IS*Ecp1-bla*_CTX-M-14_ were the second most common.

All eight *bla*_OXA-244_-positive genomes carried chromosomal ΔTn51098 variants (Table S12). In ST131-A5 (*n*=1) and ST131-A7 (*n*=6), the same IS*1R*-flanked inverted Tn1999.2 structure (ΔIS*1999-bla*_OXA-244_-Δ*lysR*) was inserted at multiple chromosomal locations. Three ST131-A7 genomes contained two or three copies of ΔTn51098. The ST131-A9 genome contained a longer ΔTn51098 element.

The *bla*_OXA-48_ gene was located on plasmids in all four genomes (Fig. 5). Three genomes carried Tn1999.2 element on pOXA-48a-like IncL plasmids (*n*=2; >99% identity and 100% coverage) or IncM1 plasmid (*n*=1; 98% identity and 90% coverage). The remaining genome carried ΔTn1999.2 element on F2:A-:B- plasmid. The IncL/IncM1 plasmids and F2:A-B- plasmid belonged to different Pling communities (Table S8).

## Discussion

Global ST131 isolates that contained O16:H5 with *fimH*41 were first reported in 2014 and named clade A by Petty and colleagues [36, 37]. When compared with other ST131 isolates (e.g. O25b:H4/clade C), ST131-A tested sensitive to the fluoroquinolones and extended-spectrum cephalosporins (e.g. ceftriaxone, ceftazidime, cefepime) [37]. Those initial studies identified a limited number of fluoroquinolone-resistant isolates that contained different combinations of QRDR mutations [36, 37]. One ST131-A isolate from this period contained *bla*_CTX-M-3_ [36]. Subsequent reports described ST131-A with various CTX-M genes, but until 2020, such genes remained rare within this clade [7, 10]. Our dataset showed that contemporary ST131-A contains multiple QRDR mutation profiles and frequently carries CTX-M genes, with marked subclade- and cluster-specific differences in their distribution (Tables 1–3). Our phylogenetic analysis revealed substantial population structure within ST131-A, with five dominant subclades accounting for 93% of genomes and ten frequent clusters accounting for 83%. This framework helped resolve lineage-specific AMR and geographic patterns, suggesting that the global expansion of ST131-A has involved multiple distinct evolutionary and epidemiological trajectories.

The population structure inferred by BAPS was generally supported by bootstrap co-clustering and maximum-likelihood phylogenetic analyses, although the strength of support varied among groups and analytical frameworks. Within-group fastbaps bootstrap values quantified the reproducibility of genome co-clustering, whereas the two maximum-likelihood branch-support measures assessed support for the MRCA node of each group. BEAST posterior probabilities and the recovery of groups as monophyletic or locally coherent components provided additional evidence regarding node support and topological coherence in the time-scaled phylogeny. The time-scaled phylogeny provided a broadly consistent picture: most dominant subclades and frequent clusters had strongly supported MRCA nodes and were recovered as monophyletic groups. In contrast, 33_A9 had low posterior support for its MRCA node, while 17_A4 and 49_A7 were represented by a small number of locally coherent components; all three clusters also had weak maximum-likelihood branch support. Thus, the BAPS-defined cluster assignments were broadly reproducible, but the precise branching relationships involving these three clusters should be interpreted with greater caution.

Clade A was previously estimated to have emerged in the mid-to-late 1800s [16]. In the mid-1900s (around the 1950s), O16:H5 was replaced with O25b:H4 and ST131-A also underwent recombination in type 1 pili from *fimH*41 to *fimH*22 to create ST131-B [16, 17]. During the late 1980s to mid-1990s, ST131-B transformed into ST131-C via a change in type 1 pili from *fimH*22 to *fimH*30 and by acquiring the following AMR determinants [10, 16]: (i) QRDR mutations in a stepwise fashion: first *parE* I529L, followed by *gyrA* S83L, *parC* S80I, *gyrA* D87N and *parC* E84V in the late 1980s to early 1990s; (ii) Some of C1 subclade gained F1:A2:B20 type plasmids with *bla*_CTX-M-14_ during the late 1990s and early 2000s; (iii) Most of ST131-C2 acquired F2:A1:B- type plasmids containing *bla*_CTX-M-15_ in early 2000s; (iv) In the late 2000s ST131-C1 obtained the prophage M27PP1 to become ST131-C1_M27 that was accompanied by a point mutation in *bla*_CTX-M-14_ to become *bla*_CTX-M-27_ [38]. Carbapenemase genes were acquired by ST131-C in the late 2000s and early 2010s [18]. The stepwise acquisition of fluoroquinolone resistance (in the 1980s), then CTX-M genes (in the 1990s), followed by carbapenemase genes (in the 2000s), is shared with other high-risk clones, such as *E. coli* ST410 [39] and *Klebsiella pneumoniae* ST307 and ST147 [40]. Our Bayesian analysis estimated the MRCA of the sampled ST131-A population to date to the 1930s, substantially later than the 1850s estimate reported in an earlier analysis of a broader ST131 dataset with greater representation of clades B and C [16]. The temporal analysis was supported by a modest but positive root-to-tip temporal signal, concordance between the TempEst slope and the estimated substitution rate, preference for the strict clock model over the optimized relaxed clock model by path-sampling marginal likelihood comparison and convergence across three independent runs. Nevertheless, the difference from the earlier estimate may reflect variation in lineage representation, genome sampling and evolutionary models.

As summarized in Table 4, the major ST131-A clusters associated with CTX-M and/or OXA-48-like genes appear to have expanded through different molecular epidemiological pathways involving chromosomal integration, plasmid carriage and clonal spread. Clusters 19_A6 and 47_A7 were the two major *bla*_CTX-M-27_-associated clusters and both diversified during the early 2000s. However, their genetic contexts differed markedly: in 19_A6, *bla*_CTX-M-27_ was consistently integrated at the same chromosomal site within a conserved structure, whereas in 47_A7 it was commonly carried by F1:A2:B20 plasmids. These contrasting genetic contexts suggest that the global expansion of *bla*_CTX-M-27_-associated ST131-A occurred through at least two different routes: clonal expansion of one lineage with conserved chromosomal integration and of another carrying a conserved plasmid backbone. *bla*_CTX-M-14_ and *bla*_CTX-M-15_ showed a different molecular epidemiological pattern from *bla*_CTX-M-27_. Both were associated with multiple globally distributed clusters that diversified during the 1990s. The two major *bla*_CTX-M-14_-associated A4 clusters differed in their QRDR profiles, indicating expansion across distinct fluoroquinolone-resistance backgrounds within the same subclade. Among complete genomes, *bla*_CTX-M-14_ was embedded in IS*Ecp1*/IS*903D*-associated elements but occurred at multiple chromosomal insertion sites and on different plasmid backbones. Molecular epidemiological inference for 33_A9 was limited by the small number of complete genomes and weak support in both the maximum-likelihood and time-scaled phylogenies. A similarly heterogeneous pattern of genetic contexts was observed for *bla*_CTX-M-15_ which was associated with major clusters in both A5 and A7. Related IS*Ecp1-bla*_CTX-M-15_-*orf477*-like elements were inserted at different chromosomal sites across these lineages and were also carried by several plasmid backbones. The combination of related *bla*_CTX-M-14_ and *bla*_CTX-M-15_ elements may have been introduced or mobilized repeatedly across ST131-A lineages, accompanied by lineage-specific clonal expansion. This contrasts with the tighter lineage and genetic-context associations of *bla*_CTX-M-27_. However, this difference may partly reflect the more recent diversification of these *bla*_CTX-M-27_-associated clusters and may, therefore, represent different stages of an ongoing evolutionary process.

**Table 4.** Characteristics of major ST131-A clusters associated with major CTX-M and OXA-48-like genes

Cluster	Associated β-lactamase, % of genomes carrying the gene	Cluster MRCA (95% HPD)/earliest β-lactamase-positive isolate, year*	QRDR background	Geographic pattern†	Gene location/genetic context‡
19_A6	*bla*_CTX-M-27_, 99%	2002 (2000–2004)/2011	*gyrA* S83L	Global (New Zealand, Australia, Japan and USA)	Consistent chromosomal integration at the same site within a conserved IS*Ecp1–bla*_CTX-M-27_–ΔIS*903D* structure
47_A7	*bla*_CTX-M-27_, 93%	2005 (2003–2006)/2013	*gyrA* S83L, D87N; *parC* S80I (E84V in subsets)	Global (USA and Australia)	Commonly carried on closely related F1:A2:B20 plasmids
21_A5	*bla*_CTX-M-15_, 33%; *bla*_OXA-244_ in a subset, 7%	1994 (1992–1995)/2006; 2021	*gyrA* S83L; additional QRDR mutations in subsets	Global (France); European countries (Denmark, Germany and Netherlands)	*bla*_CTX-M-15_ in related IS*Ecp1*/*orf477* modules at different chromosomal sites and on several plasmids; *bla*_OXA-244_ chromosomally integrated in ΔTn51098
49_A7	*bla*_CTX-M-15_, 97%; *bla*_OXA-244_ in a subset, 35%	1998 (1997–2000)/2012; 2019	*gyrA* S83L; additional QRDR mutation profiles in subsets	Global (the USA and France); European countries (Ireland and France)	*bla*_CTX-M-15_ in related IS*Ecp1*/*orf477* modules at different chromosomal sites; *bla*_OXA-244_ chromosomally integrated in ΔTn51098 at multiple sites
48_A7	*bla*_CTX-M-15_, 60%	1998 (1997–1999)/2015	*gyrA* S83L, D87N; *parC* S80I, E84V	Global (USA and Australia)	Related IS*Ecp1*/*orf477* modules in heterogeneous chromosomal and plasmid contexts
16_A4	*bla*_CTX-M-14_, 47%	1995 (1994–1997)/2009	*gyrA* S83L, D87N; *parC* S80I	Global (Australia and Japan)	Related IS*Ecp1*/IS*903D*-associated elements at multiple chromosomal sites and on plasmids
17_A4	*bla*_CTX-M-14_, 36%	1994 (1993–1996)/2006	*gyrA* S83L; additional QRDR mutations in subsets	Global (Japan)	Related IS*Ecp1*/IS*903D*-associated elements in chromosomal and different plasmid contexts
33_A9	*bla*_CTX-M-14_, 69%	1992 (1991–1994)/2007	*gyrA* S83L in minority of genomes (12%)	Global (New Zealand, Australia)	Insufficient complete-genome representation for detailed characterization
31_A9	*bla*_OXA-48_, 91%	1998 (1996–2001)/2021	Absent	Ireland	IncL pOXA-48a-like plasmid backbone sequence (short-read inference)

*Cluster MRCA dates represent the estimated diversification times of the sampled clusters. The earliest β-lactamase gene-positive isolate refers to the earliest isolate identified in the present dataset and not necessarily the first occurrence of the determinant.

†Countries accounting for >10% of genomes within each cluster carrying the specified β-lactamase are listed.

‡Gene locations and genetic contexts were assessed using complete genomes.

HPD, highest posterior density interval; MRCA, most recent common ancestor; QRDR, Quinolone Resistance-Determining Region.

OXA-48-like carbapenemases belong to three groups (A, B and C) with group A, that consists of OXA-48, OXA-244 and OXA-162, being the most common enzymes [41]. Point mutations in *bla*_OXA-48_ created *bla*_OXA-244_ within the same mobile genetic platforms (e.g. Tn*1999* on IncL plasmids) and these genes then spread independently among the *Enterobacterales,* especially *E. coli* [13]. Certain high-risk MDR *E. coli* clones are linked with the global dispersion of *bla*_OXA-48_ (e.g. ST38) and *bla*_OXA-244_ (e.g. ST38, ST69) [13]. Although OXA-48-like carbapenemases are scarce among ST131 [18], in 2024, the European Centre for Disease Prevention and Control identified two outbreaks due to ST131 with OXA-48-like carbapenemases [12]: (i) Genomes that contain *bla*_OXA-244_ and *bla*_CTX-M-15_ linked with ST131-A mainly from France, Denmark and Netherlands, (ii) Genomes that contain *bla*_OXA-48_ linked with ST131-C (and ST131-A to a lesser extent) from France and Ireland. Our results suggest different molecular epidemiological patterns for *bla*_OXA-48_ and *bla*_OXA-244_ within ST131-A. *bla*_OXA-48_ was associated with a pOXA-48a-like plasmid backbone across multiple ST131-A subclades, suggesting a role of plasmid-mediated dissemination. Within 31_A9, all *bla*_OXA-48_-positive genomes carried this backbone and were predominantly identified in Ireland, a pattern compatible with geographically concentrated clonal expansion. In contrast, *bla*_OXA-244_ was associated with at least two *bla*_CTX-M-15_-associated clusters, which showed different country-level distributions within Europe. Among complete genomes, *bla*_OXA-244_ was chromosomally integrated within ΔTn51098 variants at multiple sites in both clusters. The phylogenetic distribution of *bla*_OXA-244_-positive isolates within established *bla*_CTX-M-15_-associated lineages, together with their similar *bla*_CTX-M-15_ genetic contexts, is consistent with the subsequent introduction of *bla*_OXA-244_ followed by clonal expansion (Fig. 1 and Table S12). Overall, the recent spread of OXA-48-like carbapenemases in ST131-A involved three cluster-specific clonal expansions, each showing distinct geographic concentrations, together with additional *bla*_OXA-48_ dissemination across multiple subclades. The underlying genetic routes differed: *bla*_OXA-48_ was predominantly associated with plasmid backbones, whereas *bla*_OXA-244_ was chromosomally integrated in two separate clusters through a similar transposon-associated mechanism.

The diversity of QRDR mutation profiles within ST131-A is consistent with multiple stepwise evolutionary trajectories after subclade and cluster diversification (Fig. 1 and Table 4). Whereas *gyrA* S83L occurred across several major subclades, additional substitutions in *gyrA* D87 and *parC* S80/E84 were restricted to particular clusters or subsets within clusters, suggesting independent acquisition and differential retention under potentially distinct selective and genetic backgrounds. This contrasts with the more uniform QRDR profile of ST131-C [10, 16], in which a characteristic combination of fluoroquinolone resistance-associated mutations became fixed before clonal expansion. Distinguishing the contributions of antimicrobial selection, lineage-specific genetic backgrounds and mutation-associated fitness effects will require longitudinal genomic and epidemiological data together with experimental fitness analyses [42].

Previous studies have linked the successful expansion of ST131 clade C to the acquisition and stable maintenance of specific AMR-associated IncF plasmids, particularly F1:A2:B20 in C1-M27 and F2:A1:B- in C2, whereas F29:A-:B10 plasmids have been more commonly associated with clades A and B [5, 43]. In our ST131-A collection, F29:A-:B10 was the most common FAB profile but accounted for less than half of the genomes, and FAB profiles showed marked subclade-specific distributions (Table 2). Notably, 47_A7 carried *bla*_CTX-M-27_ on F1:A2:B20 plasmids closely related to the plasmid associated with C1-M27. This finding suggests cross-clade dissemination and subsequent establishment in ST131-A of an AMR-associated plasmid backbone also associated with clade C. The recent increase of major AMR-associated ST131-A clusters may reflect lineage-specific compatibility with particular AMR-associated plasmids, fitness costs, compensatory adaptation and ecological selection, all of which can influence the acquisition and stable maintenance of particular plasmids [44, 45]. However, many of the *bla*_CTX-M_ and *bla*_OXA-48_-like genes examined in this study were chromosomally integrated (Table 4), indicating that the expansion of AMR-associated lineages cannot be attributed solely to the acquisition and maintenance of resistance plasmids.

The increasing relative representation of A5–A7 and decreasing representation of A4 and A9 (Fig. 2a) highlight the need for continued genomic surveillance. However, because our dataset was not designed to estimate population prevalence, these patterns may reflect both the expansion of successful AMR-associated clusters within A5–A7 and temporal changes in geographic and source sampling. Prospective, geographically representative sequencing of consecutively collected ST131 isolates with denominator data and standardized metadata is needed to assess true changes in subclade and cluster frequencies.

A strength of this study is the comprehensive analysis of all publicly available *E. coli* ST131-A genomes from the NCBI databases, combined with maximum-likelihood, clustering, time-scaled Bayesian and complete-genome analyses. This approach enabled us to resolve fine-scale population structure, estimate the temporal diversification of major lineages and identify multiple molecular epidemiological patterns involving local and international clonal expansion, chromosomal integration and plasmid carriage with heterogeneous mobile genetic elements and geographic distributions. The robustness of these inferences was assessed using complementary approaches, including bootstrap co-clustering, two maximum-likelihood branch-support measures, path-sampling comparison of clock models, posterior node support and convergence diagnostics across independent Bayesian runs. However, the breadth of the dataset should not be equated with uniform global representation, because publicly available genomes disproportionately originate from countries and regions with established whole-genome sequencing capacity [46]. An additional potential source of selection bias was the exclusion of genomes with incomplete year or country metadata. Consequently, geographic differences in the frequency or apparent absence of particular subclades and clusters may reflect sampling intensity rather than their true distribution. In addition, temporal estimates remain conditional on the sampled genomes, recombination filtering and evolutionary models, and several groups showed weaker phylogenetic support. Finally, complete-genome data were available for only ∼200 genomes; therefore, inferences regarding mobile genetic structure and chromosomal integration of β-lactamase genes should be considered as representative of the analysed complete-genome subset rather than of all ST131-A genomes.

## Conclusion

Our genomic analyses indicate that the sampled MRCAs of the dominant ST131-A subclades dated mainly to the 1990s and that several MDR-associated clusters subsequently diversified during the 2000s, with diverse QRDR mutation profiles and CTX-M and OXA-48-like gene associations. Although the AMR evolution of ST131-A shared some features with that of ST131-C, the distribution and genetic contexts of *bla*_CTX-M-14_, *bla*_CTX-M-15_, *bla*_CTX-M-27,_ *bla*_OXA-48_ and *bla*_OXA-244_ differed substantially among ST131-A clusters across geographic regions. Recent European dissemination of *bla*_OXA-48_ and *bla*_OXA-244_ involved distinct ST131-A clusters with different genetic backgrounds. The identification of MDR ST131-A subclades with divergent molecular architectures emerging independently across regions highlights the challenges for public health surveillance and control efforts.

## Supplementary material

10.1099/mgen.0.001858 Supplementary Material 1.

10.1099/mgen.0.001858 Supplementary Data Sheet 1.

## Abbreviations

AMR: antimicrobial resistance
BAPS: Bayesian analysis of population structure
BSI: bloodstream infection
FAB: IncF F:A:B formula
HPD: highest posterior density
MDR: multidrug-resistant
MLST: multilocus sequence typing
MRCA: most recent common ancestor
QRDR: quinolone resistance-determining region
SH-aLRT: Shimodaira–Hasegawa-like approximate likelihood ratio test
SNP: single-nucleotide polymorphism
UFBoot: ultrafast bootstrap
VAF: virulence-associated factor
