# Supplementary material for: Genomic global epidemiology and evolution of Escherichia coli ST131 clade A
Source: Microb Genom. 2026 Oct 7;12(10):001858. doi: 10.1099/mgen.0.001858 (PMC13645330; doi:10.1099/mgen.0.001858)
Supplement: Supplementary file 1 [file mgen-12-01858-s001.pdf]

Table S1. ST131 clade A genome data sources and inclusion processes.

Dataset step	Data source			
	**NCBI Assembly (complete genome only)**	**AllTheBacteria (August 2024 update)**	**Previous studies**	**This study**
Retrieval date	31 December 2024	20 July 2025	24 April 2025	-
Genomes obtained, n	154	3643	334	97
Excluded because year was unavailable[a], n	1	94	3	0
Excluded because country was unavailable[b], n	0	5	0	0
Excluded because both year and country were unavailable	0	1104	0	0
Excluded due to quality control criteria	0	105	10	1
Final dataset, n	153[c]	2335	321	96[c]

[a] The 98 genomes with available country but unavailable year metadata were distributed across Europe/UK (n=54: Netherlands, n=36; Germany, n=10; Ireland, n=3; France, n=2; Spain, n=2; United Kingdom, n=1), the Americas (n=21: USA, n=20; Brazil, n=1), Asia/Pacific (n=13: Viet Nam, n=5; Japan, n=4; China, n=4), and other regions (n=10: Lebanon, n=5; Ghana, n=2; Nigeria, n=2; United Arab Emirates, n=1).

[b] All five genomes were collected in 2014 (n=5).

[c] The final dataset included 178 complete genomes, of which 153 were obtained from NCBI Assembly and 25 were sequenced in this study.

Table S2. Summary of fastbaps bootstrap support, phylogenetic node support, temporal estimates, and BEAST-tree component structure for ST131-A subclades and clusters.

Subclade or cluster[a]	No. of genomes (% of total)	Mean/median within-group fastbaps bootstrap value[b]	SH-aLRT/UFBoot support of MRCA node[c]	Posterior probability of the MRCA node[d]	BEAST MRCA[e] (95% HPD)	No. of BEAST tree components/singleton tips[f]	BEAST monophyly[g]
A4	357 (12%)	61.2/61	68.9/71	1	1994 (1993–1996)	1/0	Yes
16_A4	196 (7%)	97.3/100	94.6/74	1	1995 (1994–1997)	1/0	Yes
17_A4	161 (6%)	96.0/100	0/47	1	1994 (1993–1996)	2/1	No (coherent)
A5	840 (29%)	49.2/48	72.6/100	0.997	1994 (1992–1995)	1/0	Yes
21_A5	840 (29%)	49.2/48	72.6/100	0.997	1994 (1992–1995)	1/0	Yes
A6	567 (20%)	74.4/100	93.2/100	1	1998 (1997–2000)	1/0	Yes
18_A6	75 (3%)	99.5/99	99.2/100	1	2002 (2000–2003)	1/0	Yes
19_A6	489 (17%)	97.4/100	95.5/100	1	2002 (2000–2004)	1/0	Yes
20_A6	3 (<1%)	100/100	0/71	0.355	1998 (1997–2000)	1/0	Yes
A7	543 (19%)	30.5/5	0/43	0.690	1992 (1989–1994)	1/0	Yes
47_A7	150 (5%)	99.9/100	99.9/100	1	2005 (2003–2006)	1/0	Yes
48_A7	119 (4%)	97.9/100	82.1/96	1	1998 (1997–1999)	1/0	Yes
49_A7	205 (7%)	92.9/100	0/89	1	1998 (1997–2000)	2/0	No (coherent)
50_A7	2 (<1%)	100/100	99.8/100	1	2000 (1997–2002)	1/0	Yes
51_A7	2 (<1%)	100/100	87.2/100	1	1998 (1996–2001)	1/0	Yes
52_A7	14 (<1%)	97.9/100	97/100	1	1996 (1994–1998)	1/0	Yes
53_A7	19 (<1%)	96.6/100	87.7/100	1	1998 (1996–1999)	1/0	Yes
54_A7	24 (<1%)	98.9/100	76.6/100	1	1998 (1997–2000)	1/0	Yes
55_A7	6 (<1%)	100/100	0/28	0.199	1992 (1989–1994)	1/0	Yes
A9	396 (14%)	52.2/34	82.3/100	1	1992 (1991–1994)	1/0	Yes
28_A9	3 (<1%)	100/100	100/100	1	2008 (2005–2011)	1/0	Yes
29_A9	66 (2%)	98.0/100	95.7/100	1	1999 (1998–2001)	1/0	Yes
30_A9	38 (1%)	96.5/100	73.7/100	1	1998 (1996–1999)	1/0	Yes
31_A9	23 (<1%)	97.5/100	76.2/100	1	1998 (1996–2001)	1/0	Yes
32_A9	60 (2%)	96.3/100	0/53	1	1995 (1993–1996)	3/1	No (coherent)
33_A9	98 (3%)	96.4/100	0/89	0.238	1992 (1991–1994)	1/0	Yes
34_A9	25 (<1%)	95.0/96	92.5/100	1	1997 (1995–1998)	1/0	Yes
35_A9	44 (2%)	80.6/84	85.7/97	0.820	1996 (1994–1998)	1/0	Yes
36_A9	32 (1%)	96.8/98	0/23	NA	NA	2/0	No (intermixed)
37_A9	4 (<1%)	100/100	0/11	NA	NA	1/2	No (intermixed)
38_A9	2 (<1%)	99.0/99	0/23	NA	NA	0/2	No (intermixed)
A1	23 (<1%)	94.1/100	98.9/100	1	2002 (2000–2004)	1/0	Yes

Subclade or cluster[a]	No. of genomes (% of total)	Mean/median within-group fastbaps bootstrap value[b]	SH-aLRT/UFBoot support of MRCA node[c]	Posterior probability of the MRCA node[d]	BEAST MRCA[e] (95% HPD)	No. of BEAST tree components/singleton tips[f]	BEAST monophyly[g]
7_A1	18 (<1%)	100/100	99.9/100	1	2009 (2006–2011)	1/0	Yes
8_A1	2 (<1%)	100/100	95.5/100	1	2005 (2003–2007)	1/0	Yes
9_A1	2 (<1%)	99.0/99	0/29	NA	NA	0/2	No (intermixed)
A2	3 (<1%)	100/100	100/100	1	2007 (2004–2009)	1/0	Yes
5_A2	3 (<1%)	100/100	100/100	1	2007 (2004–2009)	1/0	Yes
A3	3 (<1%)	100/100	100/100	1	1998 (1996–2002)	1/0	Yes
6_A3	3 (<1%)	100/100	100/100	1	1998 (1996–2002)	1/0	Yes
A8	57 (2%)	100/100	100/100	1	1994 (1993–1996)	1/0	Yes
1_A8	2 (<1%)	100/100	100/100	1	2014 (2013–2015)	1/0	Yes
2_A8	9 (<1%)	100/100	0/93	0.491	2008 (2007–2010)	1/0	Yes
3_A8	33 (1%)	100/100	100/100	1	1994 (1993–1996)	1/0	Yes
4_A8	13 (<1%)	94.6/100	100/99	1	2005 (2003–2007)	1/0	Yes
A10	7 (<1%)	98.0/97	96.2/100	1	2000 (1998–2002)	1/0	Yes
11_A10	2 (<1%)	100/100	96.6/100	1	2014 (2013–2014)	1/0	Yes
12_A10	2 (<1%)	100/100	98/100	1	2004 (2003–2004)	1/0	Yes
A11	10 (<1%)	98.6/99	87.5/100	1	1993 (1990–1995)	1/0	Yes
40_A11	6 (<1%)	100/100	99.6/100	1	2004 (2003–2005)	1/0	Yes
41_A11	2 (<1%)	100/100	99.9/100	1	2005 (2001–2007)	1/0	Yes
A12	20 (<1%)	99.4/100	0/92	0.754	1991 (1989–1993)	1/0	Yes
44_A12	2 (<1%)	100/100	99.9/100	1	1999 (1997–2003)	1/0	Yes
45_A12	3 (<1%)	99.3/99	0/45	NA	NA	1/1	No (intermixed)
46_A12	15 (<1%)	99.9/100	0/60	NA	NA	1/1	No (intermixed)
A13	11 (<1%)	95.1/93	0/29	NA	NA	1/1	No (intermixed)
22_A13	2 (<1%)	100/100	100/100	1	2016 (2015–2016)	1/0	Yes
23_A13	2 (<1%)	100/100	100/100	1	2012 (2009–2014)	1/0	Yes
24_A13	4 (<1%)	100/100	100/100	1	2005 (2003–2006)	1/0	Yes
A14	4 (<1%)	100/100	97.3/100	1	1989 (1987–1992)	1/0	Yes
58_A14	4 (<1%)	100/100	97.3/100	1	1989 (1987–1992)	1/0	Yes
A15	9 (<1%)	100/100	91.3/100	1	1986 (1984–1988)	1/0	Yes
59_A15	2 (<1%)	100/100	100/100	1	2004 (2002–2006)	1/0	Yes
60_A15	5 (<1%)	100/100	0/94	0.435	1986 (1984–1988)	1/0	Yes
61_A15	2 (<1%)	100/100	0/92	1	1986 (1984–1988)	0/2	No (coherent)
A16	22 (<1%)	100/100	94.6/100	1	1988 (1985–1991)	1/0	Yes
62_A16	2 (<1%)	100/100	100/100	1	2006 (2005–2006)	1/0	Yes

Subclade or cluster[a]	No. of genomes (% of total)	Mean/median within-group fastbaps bootstrap value[b]	SH-aLRT/UFBoot support of MRCA node[c]	Posterior probability of the MRCA node[d]	BEAST MRCA[e] (95% HPD)	No. of BEAST tree components/singleton tips[f]	BEAST monophyly[g]
63_A16	2 (<1%)	100/100	100/100	1	2015 (2014–2015)	1/0	Yes
64_A16	5 (<1%)	100/100	100/100	1	2005 (2004–2007)	1/0	Yes
65_A16	13 (<1%)	100/100	0/49	1	1990 (1987–1993)	2/1	No (coherent)
A17	9 (<1%)	100/100	100/100	1	1964 (1960–1969)	1/0	Yes
66_A17	2 (<1%)	100/100	100/100	1	2008 (2005–2011)	1/0	Yes
67_A17	2 (<1%)	100/100	100/100	1	2012 (2011–2012)	1/0	Yes
68_A17	3 (<1%)	100/100	100/100	1	1994 (1992–1996)	1/0	Yes
69_A17	2 (<1%)	100/100	100/100	1	1979 (1975–1984)	1/0	Yes
A18	2 (<1%)	100/100	93.8/100	1	1987 (1984–1992)	1/0	Yes
70_A18	2 (<1%)	100/100	93.8/100	1	1987 (1984–1992)	1/0	Yes
A19	19 (<1%)	100/100	100/100	1	1985 (1982–1988)	1/0	Yes
71_A19	2 (<1%)	100/100	100/100	1	2020 (2019–2020)	1/0	Yes
72_A19	7 (<1%)	100/100	95.1/100	1	1995 (1993–1997)	1/0	Yes
73_A19	4 (<1%)	100/100	0/40	0.243	1985 (1982–1988)	1/0	Yes
74_A19	4 (<1%)	100/100	0/89	0.998	1987 (1984–1989)	1/1	No (coherent)

MRCA, most recent common ancestor; NA, not applicable or not assigned.

[a] Four singleton subclades (A20, A21, and A22) and 18 singleton clusters were excluded because within-group bootstrap support, group-level MRCA estimates, and component-based monophyly assessments were not applicable.

[b] Mean/median fastbaps bootstrap values represent within-group pairwise co-clustering frequencies using the default settings of boot_fast_baps.

[c] Shimodaira–Hasegawa-like approximate likelihood ratio test (SH-aLRT) and ultrafast bootstrap (UFBoot) values represent local branch support for the maximum-likelihood node defining the group. Both were estimated using 1,000 replicates in IQ-TREE. Low node support indicates uncertainty in the exact local branching pattern.

[d] All BEAST-derived estimates were obtained under the strict clock model (Table S5).

[e] MRCA estimates were reported for monophyletic groups and for groups classified as "No (coherent)." They were not reported for "No (intermixed)" groups because the common ancestral node subtending all assigned genomes was not considered representative of the group. For "No (coherent)" groups, the MRCA node also included genomes assigned to other groups.

[f] A component was defined as a maximal monophyletic group containing at least two genomes assigned to the same subclade or cluster. Singleton tips were counted separately.

[g] Groups classified as "No (coherent)" were represented by multiple components and/or singleton tips that remained locally grouped in the BEAST tree, whereas "No (intermixed)" indicated broader interspersion with genomes assigned to other clusters.

Table S3. Virulence profiles associated with different dominant ST131 A subclades.

Gene	A4<br>n=357 (%)	A5<br>n=840 (%)	A6<br>n=567 (%)	A7<br>n=543 (%)	A9<br>n=396 (%)	All<br>n=2905 (%)
Adhesions:						
papA_F43	331 (93%)	814 (97%)†	486 (86%)†	529 (97%)†	382 (96%)†	2665 (92%)
iha	336 (94%)	720 (86%)†	553 (98%)†	536 (99%)†	385 (97%)†	2663 (92%)
fimH	357 (100%)	839 (100%)	566 (100%)	543 (100%)	395 (100%)	2902 (100%)
hra	8 (2%)†	95 (11%)†	0†	24 (4%)	12 (3%)	156 (5%)
afaA	17 (5%)†	477 (57%)†	69 (12%)†	80 (15%)†	220 (56%)†	875 (30%)
Toxins						
hlyA	1 (<1%)†	25 (3%)†	443 (78%)†	6 (1%)†	1 (<1%)†	483 (17%)
sat	248 (69%)	568 (68%)†	466 (82%)†	444 (82%)†	280 (71%)	2085 (72%)
astA	2 (<1%)†	80 (10%)†	70 (12%)†	1 (<1%)†	7 (2%)†	160 (6%)
cnf1	1 (<1%)†	25 (3%)†	447 (79%)†	6 (1%)†	1 (<1%)†	487 (17%)
Siderophores						
iroN	0	0	0	3 (<1%)	1 (<1%)	7 (<1%)
fyuA	357 (100%)	837 (100%)	567 (100%)	543 (100%)	396 (100%)	2902 (100%)
ireA	3 (<1%)	13 (2%)†	2 (<1%)	1 (<1%)	0	19 (<1%)
iutA	249 (70%)	571 (68%)†	469 (83%)†	450 (83%)†	284 (72%)	2125 (73%)
Capsular antigen						
kpsM II	50 (14%)	49 (6%)†	1 (<1%)†	167 (31%)†	19 (5%)†	293 (10%)
kpsMII_K4	0	1 (<1%)	0	0	5 (1%)†	6 (<1%)
kpsMII_K5	100 (28%)†	713 (85%)†	553 (98%)†	366 (67%)†	364 (92%)†	2267 (78%)
kpsM_K15	172 (48%)†	0†	0†	0†	0†	185 (6%)
Miscellaneous						
usp	352 (99%)†	840 (100%)	567 (100%)	542 (100%)	396 (100%)	2899 (100%)

Gene	A4<br>n=357 (%)	A5<br>n=840 (%)	A6<br>n=567 (%)	A7<br>n=543 (%)	A9<br>n=396 (%)	All<br>n=2905 (%)
traT	331 (93%)	806 (96%)†	559 (99%)†	496 (91%)†	373 (94%)	2732 (94%)
ompT	357 (100%)†	838 (100%)†	567 (100%)†	531 (98%)	365 (92%)†	2859 (98%)
iss	8 (2%)	27 (3%)	1 (<1%)†	9 (2%)†	22 (6%)	100 (3%)
cvaC	0	0	0	2 (<1%)	1 (<1%)	5 (<1%)

papA, P fimbriae genes of P fimbriae operon; *iha*, iron-regulated adhesin; *fimH*, type-1 fimbriae; *hra*, heat-resistant agglutinin, *afa,* afimbrial adhesin; *hlyA*, α-hemolysin; *sat*, secreted autotransporter toxin; *vat*, vacuolating autotransporter toxin; *astA*, enteroaggregative *E. coli* toxin; *cnf1*, cytotoxic necrotizing factor; *iroN*, salmochelin (siderophore) receptor; *fyuA*, yersiniabactin (siderophore) receptor; *ireA*, iron-regulated element (catecholate siderophore); *iutA*, aerobactin (siderophore) receptor; *kps*, group II capsule variants synthesis; *usp*, uropathogenic-specific protein; *traT*, complement inhibition protein; *ompT*, outer membrane protein T; *iss*, increased serum survival; *malX*, pathogenicity island marker; *cvaC*, factor facilitating colonization

† Bonferroni-corrected $P < 0.05$ by Fisher's exact test, comparing each subclade with all remaining ST131-A genomes.

Table S4. Comparison of major CTX-M and OXA-48-like gene carriage in associated ST131-A clusters.

Gene	19_A6 n=489 (%)	47_A7 n=150 (%)	16_A4 n=196 (%)	17_A4 n=161 (%)	33_A9 n=98 (%)	21_A5 n=840 (%)	49_A7 n=205 (%)	48_A7 n=119 (%)	31_A9 n=23 (%)	All n=2905 (%)
CTX-M-27	486 (99%)†	140 (93%)†	9 (5%)†	3 (2%)†	10 (10%)†	19 (2%)†	0†	28 (24%)	0†	750 (26%)
CTX-M-15	2 (<1%)†	7 (5%)†	3 (2%)†	9 (6%)†	1 (1%)†	281 (33%)†	199 (97%)†	71 (60%)†	0	627 (22%)
CTX-M-14	3 (<1%)†	0†	92 (47%)†	58 (36%)†	68 (69%)†	29 (3%)†	0†	2 (2%)†	0	298 (10%)
OXA-244	0†	4 (3%)	0†	0†	0	59 (7%)†	71 (35%)†	0†	0	142 (5%)
OXA-48	2 (<1%)†	2 (1%)	10 (5%)	4 (2%)	4 (4%)	31 (4%)	7 (3%)	8 (7%)	21 (91%)†	125 (4%)

† Bonferroni-corrected $P < 0.05$ by Fisher's exact test, comparing each cluster with all remaining ST131-A genomes.

Table S5. BEAST model settings, marginal likelihoods, and posterior estimates.

Variable	Strict clock	Optimised relaxed clock
Path-sampling model comparison[a]		
Log marginal likelihood	-5,011,633	-5,209,949
Difference in log marginal likelihood (strict minus relaxed)[b]	198,316	
MCMC settings		
MCMC chain length per run	800 million	1 billion
Number of independent runs	3	3
Burn-in removed from each run[c]	10%	20%
Posterior estimates		
Mean clock rate[d], substitutions/site/year (95% HPD)	5.58×10^{-7} (5.39–5.76×10^{-7})	6.25×10^{-7} (5.87–6.60×10^{-7})
Mean tree height, years (95% HPD)	293 (275–312)	317 (136–537)

MCMC, Markov chain Monte Carlo; HPD, highest posterior density interval; ESS, effective sample size.

[a] Marginal likelihood estimation was performed with 100 path-sampling steps, an alpha parameter of 0.3, a chain length of 1,000,000 per step, 50% burn-in, and 1,000,000 pre-burn-in samples.

[b] The difference in log marginal likelihood (ML) was calculated as log ML_{strict} − log $ML_{relaxed}$; positive values support the strict clock model.

[c] Burn-in was determined separately for each clock model based on visual inspection of posterior traces.

[d] For the optimised relaxed clock model, the reported mean clock rate corresponds to ORCUcldMean.

Table S6. Effective sample sizes for key parameters of the BEAST models.[a]

Clock model	Parameter	Run 1	Run 2	Run 3	Combined
Strict	clockRate	406	397	409	1,206
Strict	Tree.height	1,642	1,587	1,668	4,851
Strict	bPopSizes.1	1,278	1,863	1,653	4,386
Strict	bPopSizes.2	203	634	201	602
Strict	bPopSizes.3	201	343	201	622
Strict	bPopSizes.4	587	574	645	1,735
Strict	bPopSizes.5	1011	784	983	2,706
Optimised relaxed	ORCucldMean	398	279	270	763
Optimised relaxed	ORCsigma	606	560	492	1,522
Optimised relaxed	Tree.height	6,897	8,611	8,849	23,791
Optimised relaxed	bPopSizes.1	1,243	1,326	1,097	3,071
Optimised relaxed	bPopSizes.2	627	536	508	1,231
Optimised relaxed	bPopSizes.3	286	244	280	651
Optimised relaxed	bPopSizes.4	376	315	298	747
Optimised relaxed	bPopSizes.5	516	390	411	1,010

[a] Effective sample sizes were calculated after removal of model-specific burn-in (Table S5). Combined values were obtained after combining the three independent runs following burn-in removal.

Table S7. Pling community-level summary of plasmids from ST131-A complete genomes.

Pling community	No. of plasmids	No. of subcommunities	No. of host genomes	Frequent host clusters[a] (n)	Median plasmid size, kb (range)	Replicon type/FAB profile[b] (n)	CTX-M or carbapenemase genes detected on plasmids (n)
community_0	196	44	164	21_A5 (50), 19_A6 (39), 17_A4 (13), 16_A4 (12), 47_A7 (10), 48_A7 (9), 49_A7 (9), 33_A9 (3), 29_A9 (2), 18_A6 (1), others (48)	131.4 (13.1–212.2)	F29:A-:B10 (83), F1:A2:B20 (23), F2:A-:B- (11), F29:A-:B10-like (11), F1:A1:B23 (8), F1:A1:B16 (6), F2:A-:B10 (4), untypeable (4), other profiles (46)	CTX-M-27 (16), CTX-M-14 (5), CTX-M-15 (4), NDM-5 (2), OXA-48 (1)
community_1	6	3	6	21_A5 (5), 48_A7 (1)	110.8 (108.6–111.7)	IncFIB(H89-PhagePlasmid) (6)	CTX-M-15 (5)
community_2	16	15	16	21_A5 (10), 16_A4 (2), 48_A7 (1), others (3)	86.5 (79.0–103.1)	IncI1-I(Alpha) (13), IncI(Gamma) (3)	CTX-M-15 (3); CTX-M-55, CTX-M-123 (1 each)
community_3	4	4	4	16_A4 (2), 29_A9 (1), others (1)	66.5 (58.5–74.3)	IncM1 (2), IncL (2)	OXA-48 (3)
community_4	3	3	3	17_A4, 19_A6, 21_A5 (1 each)	94.8 (94.4–94.8)	IncY (3)	Absent
community_5	7	7	7	17_A4 (3), 21_A5 (2), others (2)	90.7 (82.6–103.3)	IncB/O/K/Z (7)	CTX-M-14 (3), CTX-M-15 (1)
community_6	3	3	3	others (3)	34.4 (34.3–40.0)	Untypeable (2), IncX1 (1)	Absent
community_7	3	2	3	16_A4, 47_A7, others (1 each)	34.6 (34.5–35.0)	IncN4 (2), rep_cluster_1760 (1)	Absent
community_8	2	2	2	17_A4 (1), others (1)	56.5 (49.8–63.3)	IncN (2)	CTX-M-2, IMP-1 (1 each)
community_9	2	2	2	21_A5 (2)	125.4 (113.6–137.1)	FIIK2 (2)	KPC-3, NDM-5 (1 each)
community_10	2	1	2	49_A7 (2)	34.6 (34.6–34.6)	IncX4 (2)	Absent
Singleton communities	15	15	15	21_A5 (6), 19_A6 (2); 16_A4, 17_A4, 33_A9, 47_A7,	62.2 (23.9–275.2)	Untypeable (3); ColKP3+IncX3, F43:A-:B-, IncA, IncFIB(AP001918), IncI2, IncN2, IncP1,	CTX-M-15, KPC-3, OXA-181, VIM-1 (1 each)

Pling community	No. of plasmids	No. of subcommunities	No. of host genomes	Frequent host clusters[a] (n)	Median plasmid size, kb (range)	Replicon type/FAB profile[b] (n)	CTX-M or carbapenemase genes detected on plasmids (n)
				48_A7 (1 each), others (2)		IncX3rep_cluster_435, rep_cluster_1704, rep_cluster_2221, pKPC-CAV1193, pSL483+pXuzhou21 (1 each)	
Unassigned hub plasmids	3	NA	3	17_A4, 19_A6, others (1 each)	16.4 (15.9–37.1)	Untypeable (2), F-:A-:B20 (1)	Absent

NA, not assigned.

[a] Frequent host clusters comprised 16_A4, 17_A4, 18_A6, 19_A6, 21_A5, 29_A9, 33_A9, 47_A7, 48_A7, and 49_A7. All remaining clusters were combined as "others".

Counts are based on plasmids identified among the available complete genomes and should not be interpreted as prevalence estimates.

[b] IncF plasmids are shown using the FAB formula; non-IncF plasmids are shown by replicon type.

Table S8. Pling subcommunity-level summary of plasmids from ST131-A complete genomes.

Pling community	Pling subcommunity	No. of plasmids	No. of host genomes	Frequent host clusters[a] (n)	Median plasmid size, kb (range)	Replicon type/FAB profile[b] (n)	CTX-M or carbapenemase genes detected on plasmids (n)
community_0	subcommunity_33	2	2	47_A7 (2)	131.4 (131.4–131.4)	F2:A4:B- (2)	NDM-5 (2)
community_0	subcommunity_35	2	2	48_A7 (2)	120.4 (78.0–162.9)	F2:A2:B20, F2:A-:B- (1 each)	CTX-M-15 (2), CTX-M-27 (1)
community_0	subcommunity_36	4	4	others (4)	141.4 (105.7–146.9)	F1:A1:B23 (4)	Absent
community_0	subcommunity_37	3	3	16_A4 (2), 21_A5 (1)	86.8 (65.2–87.0)	F2:A-:B- (3)	OXA-48 (1)
community_0	subcommunity_38	3	3	48_A7 (3)	126.3 (117.1–146.8)	F1:A2:B20 (3)	Absent
community_0	subcommunity_39	4	4	49_A7 (2), 48_A7 (1), others (1)	72.9 (17.6–75.8)	F10:A-:B-, IncFII(29), IncFII(29)+IncFII(pAMA1167-NDM-5), untypeable (1 each)	Absent
community_0	subcommunity_40	4	4	17_A4, 19_A6 (1 each), others (2)	70.3 (38.0–70.3)	F2:A-:B- (2), F35:A-:B-, F59-like:A-:B- (1 each)	CTX-M-14 (3), CTX-M-27 (1)
community_0	subcommunity_41	6	6	others (6)	140.7 (66.3–142.7)	F29:A-:B10 (5), F-:A-:B10 (1)	Absent
community_0	subcommunity_42	7	7	49_A7 (7)	137.5 (88.2–141.2)	F1:A1:B16 (6), F-:A1:B16 (1)	Absent
community_0	subcommunity_43	9	9	47_A7 (6), 48_A7 (2), 21_A5 (1)	117.9 (69.3–146.9)	F1:A2:B20 (8), F1-like:A2:B20 (1)	CTX-M-27 (9)
community_0	subcommunity_44	12	12	17_A4 (6), 16_A4 (5), 47_A7 (1)	130.9 (81.1–155.3)	F1:A2:B20 (9), F-:A2:B20 (2), F1:A2:B- (1)	Absent
community_0	subcommunity_45	108	103	21_A5 (43), 19_A6 (38), 16_A4, 18_A6, 29_A9, 33_A9 (1 each), others (23)	135.8 (13.1–212.2)	F29:A-:B10 (78), F29:A-:B10-like (10), F2:A-:B10 (4), F4:A-:B-, Untypeable (3 each), F4:A-:B10 (2), other profiles (8)	CTX-M-27 (3), CTX-M-15 (1)
community_1	subcommunity_1	3	3	21_A5 (2), 48_A7 (1)	110.8 (110.8–110.8)	IncFIB(H89-PhagePlasmid) (3)	CTX-M-15 (3)

Pling community	Pling subcommunity	No. of plasmids	No. of host genomes	Frequent host clusters[a] (n)	Median plasmid size, kb (range)	Replicon type/FAB profile[b] (n)	CTX-M or carbapenemase genes detected on plasmids (n)
community_1	subcommunity_2	2	2	21_A5 (2)	111.7 (111.7–111.7)	IncFIB(H89-PhagePlasmid) (2)	CTX-M-15 (2)
community_2	subcommunity_14	2	2	21_A5 (2)	81.2 (80.1–82.2)	IncI1-I(Alpha) (2)	Absent
community_7	subcommunity_1	2	2	16_A4, 47_A7 (1 each)	34.6 (34.5–34.6)	IncN4 (2)	Absent
community_10	subcommunity_0	2	2	49_A7 (2)	34.6 (34.6–34.6)	IncX4 (2)	Absent

Only Pling subcommunities containing at least two ST131-A plasmids were included.

[a] Frequent host clusters comprised 16_A4, 17_A4, 18_A6, 19_A6, 21_A5, 29_A9, 33_A9, 47_A7, 48_A7, and 49_A7. All remaining clusters were combined as “others”.

Counts are based on plasmids identified among the available complete genomes and should not be interpreted as prevalence estimates.

[b] IncF plasmids are shown using the FAB formula; non-IncF plasmids are shown by replicon type.

Table S9. Gene location and genetic environments of *bla*$_{CTX-M-27}$

	Subclade					
	**A2**	**A5 (cluster 21)**	**A6**	**A7**	**A8**	**A9**
**Location**	**n=1**	**n=1**	**n=36**	**n=11**	**n=1**	**n=3**
Chromosome			IS*Ecp1*-*bla*$_{CTX-M-27}$-ΔIS*903D* (415 bp) inserted into *gspD*[a] (cluster 19, n=36)	ΔIS*Ecp1* (387 bp)-*bla*$_{CTX-M-27}$-IS*903D*-*iroN*-ΔTn2 (308 bp) inserted into 50S ribosome-binding GTPase gene (cluster 52, n=1)		
F1:A2:B20				ΔIS*1R* (473bp)-ΔTn2 (592bp)-IS*26*-ΔIS*Ecp1* (208 bp)-*bla*$_{CTX-M-27}$-ΔIS*903D* (391 bp)-IS*26* (cluster 47, n=6; cluster 48, n=2)		
F2:A2:B20				IS*26*-ΔIS*Ecp1* (208 bp)-*bla*$_{CTX-M-27}$-ΔIS*903D* (391 bp)-IS*26* (cluster 48, n=1)		
F2:A-:B10						ΔIS*1R* (273bp)-IS*26*-ΔIS*26* (84 bp)- ΔIS*Ecp1* (388 bp)-*bla*$_{CTX-M-27}$-IS*903D*-*iroN*-ΔTn2 (312bp)-IS*26* (cluster 32, n=1)
						ΔIS*1R* (473 bp)-ΔTn2 (592 bp)-IS*26*-ΔIS*Ecp1*

Location	Subclade A2 n=1	A5 (cluster 21) n=1	A6 n=36	A7 n=11	A8 n=1	A9 n=3
						(388 bp)-*bla*$_{CTX-M-27}$-IS*903D*-*iroN*-ΔTn2 (312 bp)-IS*26* (cluster 33, n=1)
F1:A1:B20	ΔIS1 (473 bp)-ΔTn2 (592 bp)-IS*26*-ΔIS*Ecp1* (388 bp)-*bla*$_{CTX-M-27}$-IS*903D*-*iroN*-IS*26* (cluster 5, n=1)					
F1-like:A2:B20		ΔIS1 (473 bp)-ΔTn2 (592 bp)-IS*26*-ΔIS*Ecp1* (208 bp)-*bla*$_{CTX-M-27}$-ΔIS*903D* (391 bp)-ΔTn2 (2301 bp)-ΔTn21-IS*26* (n=1)				
F29:A-:B10					IS*26*-ΔIS*Ecp1* (399 bp)-*bla*$_{CTX-M-27}$-IS*903D*-*iroN*-ΔTn2 (685 bp)-IS*26* (cluster 3, n=1)	
F35:A2:B20				IS*1R*-ΔIS*10R* (148 bp)-IS*26*-ΔIS*Ecp1* (208 bp)-*bla*$_{CTX-M-27}$-ΔIS*903D* (391 bp)-IS*26* (n=1, cluster 48)		

	**Subclade**					
	**A2**	**A5 (cluster 21)**	**A6**	**A7**	**A8**	**A9**
**Location**	**n=1**	**n=1**	**n=36**	**n=11**	**n=1**	**n=3**
F59-like:A-:B-						*eitABCD*-IS*Ecp1*-*bla*$_{CTX-M-27}$-ΔIS*903D* (484 bp)-*malB* (cluster 30, n=1)

[a] One genome had a second copy of the IS*Ecp1*-*bla*$_{CTX-M-27}$-△IS*903D* (415 bp) environment inserted into *hlyA*.

Table S10. Gene location and genetic environments of *bla*$_{\text{CTX-M-15}}$

	Subclade		
	**A4**	**A5 (cluster 21)**	**A7**
**Location**	**n=1**	**n=15**	**n=12**
Chromosome		IS*Ecp1*-*bla*$_{\text{CTX-M-15}}$-*orf477*-ΔTn2 (79 bp) inserted into an intergenic region between sugar kinase and hypothetical protein genes (n=4)<br>IS*Ecp1*-*bla*$_{\text{CTX-M-15}}$-*orf477* inserted into an intergenic region between an ISFl3-like element and a hypothetical protein gene (n=1[a])	IS*Ecp1*-*bla*$_{\text{CTX-M-15}}$-*orf477* inserted into an intergenic region between the *smrB* and *prmB* genes (cluster 49, n=6[b])<br>IS*Ecp1*-*bla*$_{\text{CTX-M-15}}$-*orf477* inserted into an intergenic region between a fimbrial protein gene and the *prmB* gene (cluster 49, n=2[c])<br>IS*Ecp1*-*bla*$_{\text{CTX-M-15}}$-*orf477*-ΔTn2 (1835 bp) inserted into an intergenic region between hypothetical protein genes (cluster 48, n=1)
IncFIB(H89-PhagePlasmid)		IS*Ecp1*-*bla*$_{\text{CTX-M-15}}$-*orf477*-ΔTn2 (87 bp) (n=3)<br>IS*Ecp1*-*bla*$_{\text{CTX-M-15}}$-*orf477* (n=1)	IS*Ecp1*-*bla*$_{\text{CTX-M-15}}$-*orf477*-ΔTn2 (87 bp) (cluster 48, n=1)
F1:A1:B66		IS*26*-ΔIS*Ecp1* (155 bp)-*bla*$_{\text{CTX-M-15}}$-*orf477*-ΔTn2 (2246 bp)-IS*26*-ΔTn2 (592 bp)-IS1 (n=1)	
F2:A-:B-			IS*26*-ΔIS*Ecp1* (159 bp)-*bla*$_{\text{CTX-M-15}}$-*orf477*-ΔTn2 (2246 bp)-IS*26* (cluster 48, n=1)
F2:A2:B20			IS*26*-ΔIS*Ecp1* (159 bp)-*bla*$_{\text{CTX-M-15}}$-*orf477*-ΔTn2 (2246 bp)-IS*26* (cluster 48, n=1)
F22,36:A1:B1	IS*1R*-ΔIS*Ecp1* (299 bp)-*bla*$_{\text{CTX-M-15}}$-*orf477*-ΔTn2 (2830 bp)-IS*26* (cluster 16, n=1)		
IncB/O/K/Z		IS*26*-ΔIS*Ecp1* (144 bp)-*bla*$_{\text{CTX-M-15}}$-*orf477* inserted into Tn2 (n=1)	

	Subclade		
Location	A4<br>n=1	A5 (cluster 21)<br>n=15	A7<br>n=12
IncI1-I(Alpha)		IS*Ecp1*-*bla*$_{CTX-M-15}$-*orf477* inserted into Tn2 (n=2)	
IncI(Gamma)		IS*Ecp1*-*bla*$_{CTX-M-15}$-*orf477* (n=1)	
IncI2		IS*Ecp1*-*bla*$_{CTX-M-15}$-*orf477* (n=1)	

[a] This genome also carried *bla*$_{OXA-244}$ on chromosome.

[b] Four out of six genomes also carried *bla*$_{OXA-244}$ on chromosome.

[c] These two genome also carried *bla*$_{OXA-244}$ on chromosome.

Table S11. Gene location and genetic environments of *bla*$_{\text{CTX-M-14}}$

	Subclade				
**Location**	**A4**<br>**n=10**	**A5 (cluster 21)**<br>**n=1**	**A6**<br>**n=1**	**A9**<br>**n=2**	**A17**<br>**n=1**
Chromosome	IS*Ecp1*-*bla*$_{\text{CTX-M-14}}$-ΔIS*903D* (484 bp)-*malB* inserted into *ghxQ* as a first copy and IS*Ecp1*-*bla*$_{\text{CTX-M-14}}$-ΔIS*903D* (484 bp)-Δ*malB* (93 bp) inserted into an intergenic region between the *yhiY* and *yhiM* genes as a second copy[a] (cluster 16, n=3)<br>IS*Ecp1*-*bla*$_{\text{CTX-M-14}}$-IS*903D*-*iroN* inserted into *yjeA* (cluster 16, n=1)<br>IS*Ecp1*-*bla*$_{\text{CTX-M-14}}$-ΔIS*903D* (484 bp)-*malB* inserted into an intergenic region between the *timP* and *sseA* genes (cluster 17, n=1)<br>IS*Ecp1*-*bla*$_{\text{CTX-M-14}}$-ΔIS*903D* (484 bp) inserted into the NRAMP family divalent metal transporter gene (cluster 17, n=1)			IS*Ecp1*-*bla*$_{\text{CTX-M-14}}$-ΔIS*903D* (446 bp) inserted into *bcsB* (cluster 33, n=1[b])	IS*Ecp1*-*bla*$_{\text{CTX-M-14}}$-ΔIS*903D* (481 bp) inserted into an intergenic region between the *alx* and *sst* genes (cluster 66, n=1)
F1:A2:B20				IS*Ecp1*-*bla*$_{\text{CTX-M-14}}$-ΔIS*903D* (446 bp) (cluster 33, n=1[b])	
F2:A-:B-	*eitABCD*-IS*Ecp1*-*bla*$_{\text{CTX-M-14}}$-ΔIS*903D* (484 bp)-*malB* (cluster 17, n=2)			*eitABCD*-IS*Ecp1*-*bla*$_{\text{CTX-M-14}}$-ΔIS*903D* (484 bp)-*malB* (cluster 34, n=1)	
F35:A-:B-			*eitABCD*-IS*Ecp1*-*bla*$_{\text{CTX-M-14}}$-ΔIS*903D*		

Location	Subclade A4 n=10	A5 (cluster 21) n=1	A6 n=1	A9 n=2	A17 n=1
			(484 bp)-*malB* (cluster 19, n=1)		
IncB/O/K/Z	IS*Ecp1*-*bla*$_{\text{CTX-M-14}}$-IS*903D*-*iroN* (cluster 17, n=1)	IS*Ecp1*-*bla*$_{\text{CTX-M-14}}$-ΔIS*903D* (484 bp) (n=1)			

[a] Two genomes contained a third copy of the IS*Ecp1*-*bla*$_{\text{CTX-M-14}}$-△IS*903D* module with lengths variations in the IS*903D* element. One had a 484-bp △IS*903D* element inserted into *flhA*, and the other had a 463-bp △IS*903D* inserted into a carbohydrate porin gene.

[b] The same IS*Ecp1*-*bla*$_{\text{CTX-M-14}}$-ΔIS*903D* (446 bp) module was carried on both the chromosome and an F1:A2:B20 plasmid.

Table S12. Gene location and genetic environments of *bla*$_{OXA-244}$

Chromosomal integration site	Subclade		
	**A5 (cluster 21) n=1**	**A7 n=6**	**A9 n=1**
Endonuclease gene	ΔTn51098[a] (IS*1R*-ΔIS*1999* [138 bp]-*bla*$_{OXA-244}$-Δ*lysR* [506 bp]-IS*1R*; n=1)	ΔTn51098[a] (IS*1R*-ΔIS*1999* [138 bp]-*bla*$_{OXA-244}$-Δ*lysR* [506 bp]-IS*1R*; cluster 49, n=2)	
5' side: endonuclease gene<br>3' side: porin family protein gene		ΔTn51098[a] (IS*1R*-ΔIS*1999* [138 bp]-*bla*$_{OXA-244}$-Δ*lysR* [506 bp]-IS*1R*; cluster 49, n=1)<br>2 copies of ΔTn51098[a] (IS*1R*-ΔIS*1999* [138 bp]-*bla*$_{OXA-244}$-Δ*lysR* [506 bp]-IS*1R*-ΔIS*1999* [138bp]-*bla*$_{OXA-244}$-Δ*lysR* [506 bp]-IS*1R*; cluster 49, n=1)	
Combination of beta-family protein and porin family protein genes		3 copies of ΔTn51098[a] (IS*1R*-ΔIS*1999* [138 bp]-*bla*$_{OXA-244}$-Δ*lysR* [506 bp]-IS*1R*): ΔTn51098[a] inserted into beta family protein, followed by two identical copies of 5 ORFs, a partial endonuclease gene, and ΔTn51098[a] (the downstream region of the final ΔTn51098[a] contained a porin family protein disrupted by the 3' side IS*1R* of ΔTn51098[a]; cluster 49, n=2)	
5' side: endonuclease gene<br>3' side: *leoA*			ΔTn51098[b] (IS*1R*-ΔIS*1999* [138 bp]-*bla*$_{OXA-244}$-*lysR*-*pemIK*-*mucAB*-*orf5*-*orf6*-*orf7*-*orf8*-Δ*orf9*; cluster 29, n=1)

ORF, open reading frame.

[a] Compared with the original Tn51098, the deletion of the *lysR* downstream region (IS*1999* and the backbone genes of pOXA-48a) resulted in the 5' portion of *lysR* being directly joined to IS*1R*, leaving IS*1R*-flanked ΔIS*1999*-*bla*$_{OXA-244}$-Δ*lysR* structure.

[b] Compared with the original Tn51098, all genes from the internal portion of *orf9* onward (the backbone genes of pOXA-48a and IS*1R*) were deleted.

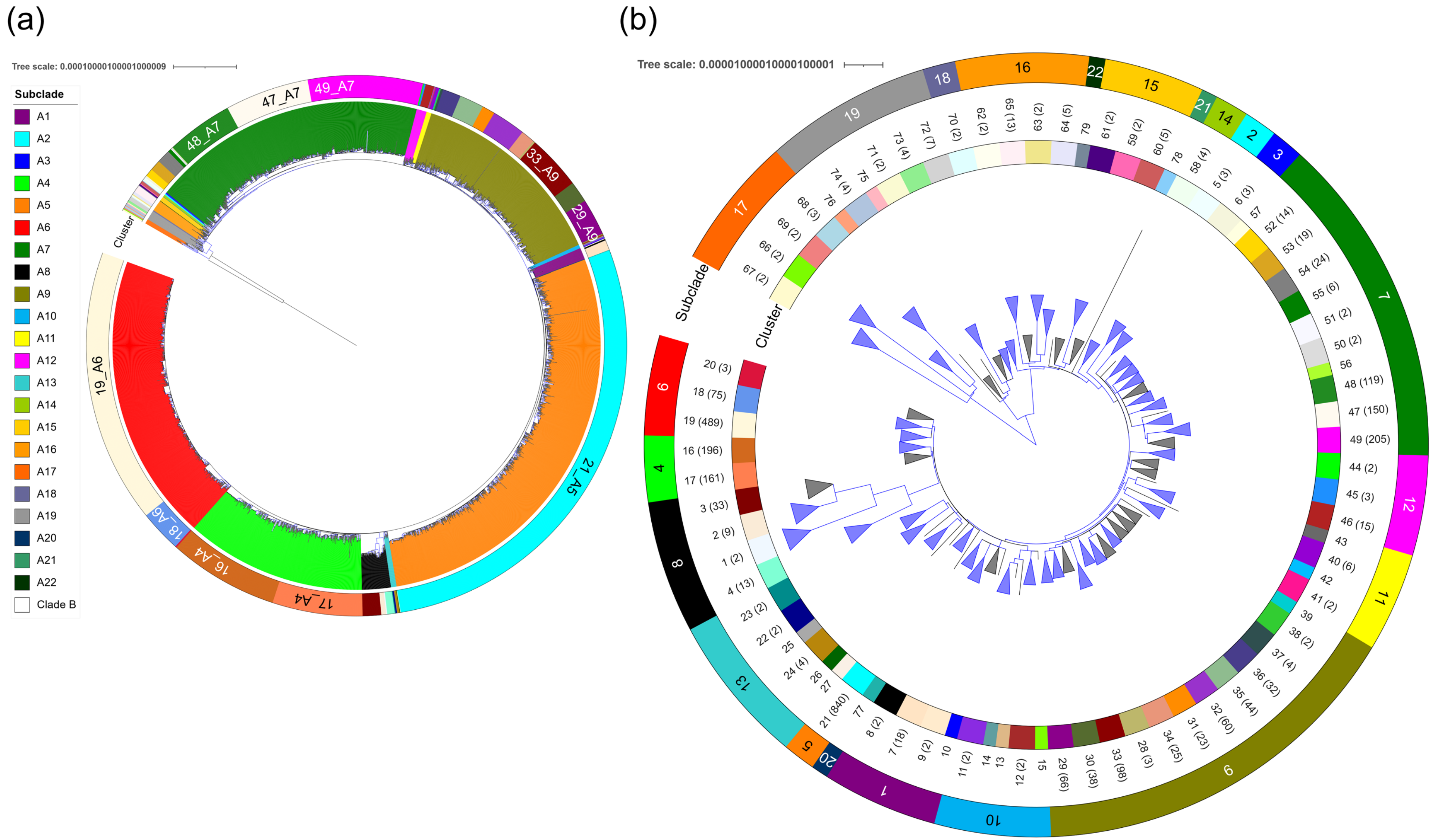

Figure S1. Full and collapsed representations of the maximum-likelihood phylogeny of ST131-A genomes.

(a) Full maximum-likelihood phylogeny of 2,905 ST131-A genomes, including the clade B isolate SaT040 used as the outgroup. Subclades are indicated by coloured shading within the phylogram. Frequent clusters are labelled within the cluster annotation strip using the cluster number followed by the corresponding subclade designation (e.g. 16_A4).

(b) Enlarged and collapsed representation of the ST131-A portion of the same phylogeny after pruning the clade B outgroup for visualisation. Each monophyletic cluster is collapsed, with the number of genomes represented shown in parentheses. Branches leading to labelled groups are coloured

blue when the corresponding MRCA node received both SH-aLRT ≥80% and UFBoot ≥95% support. Numerical support values are provided in Table S1. Numbers in the subclade color strip denote A1–A22; the prefix "A" is omitted for clarity.

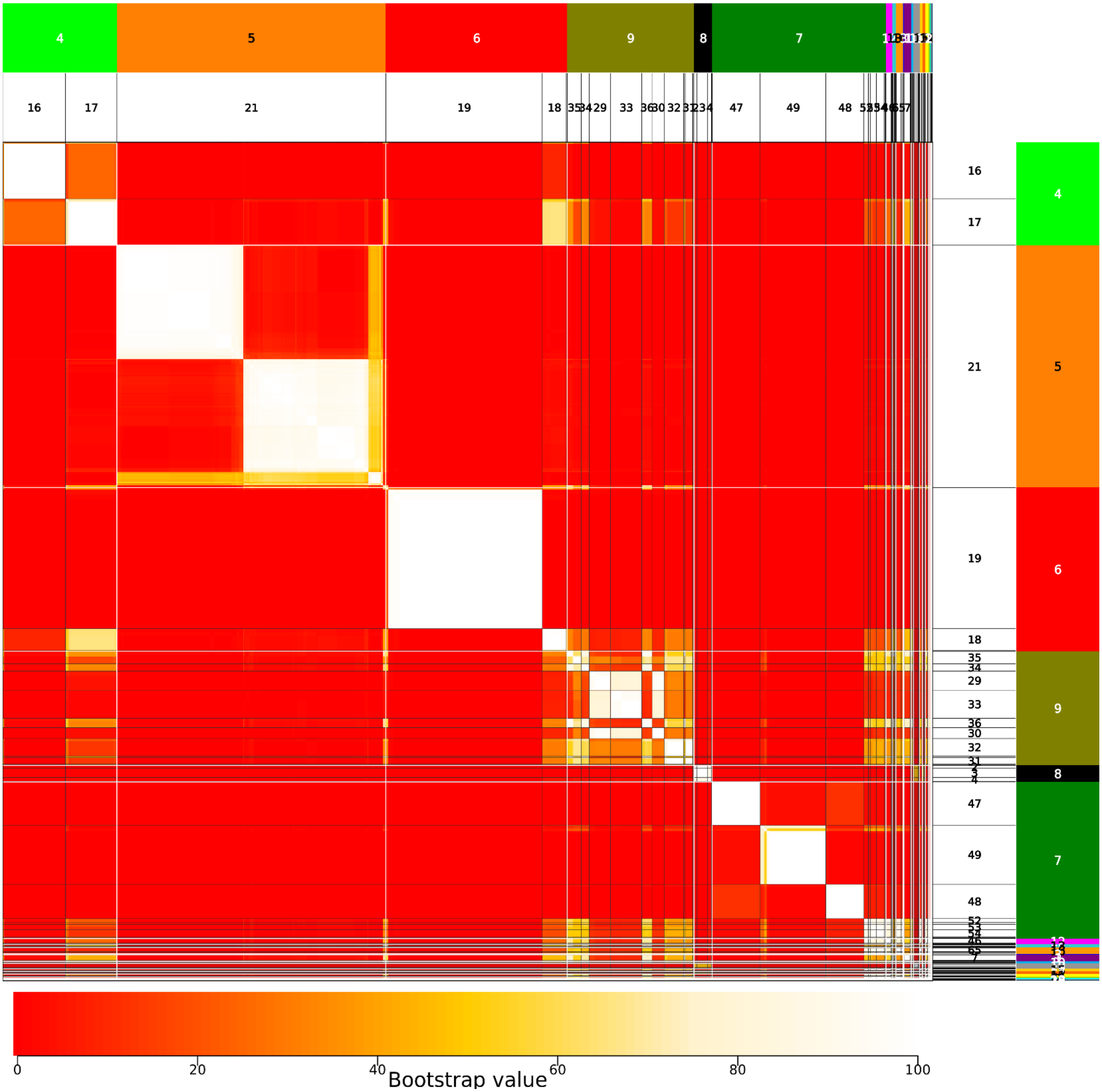

Figure S2. Fastbaps bootstrap co-clustering heatmap.

Pairwise bootstrap co-clustering values are shown after ordering isolates by subclades (BAPS level 1) and clusters (BAPS level 2). The upper and right annotation bars indicate subclades, and internal labels indicate clusters. Higher values indicate more frequent co-clustering across bootstrap replicates. Subclade colours are harmonised with those used for subclades in Figures 1 and 2.

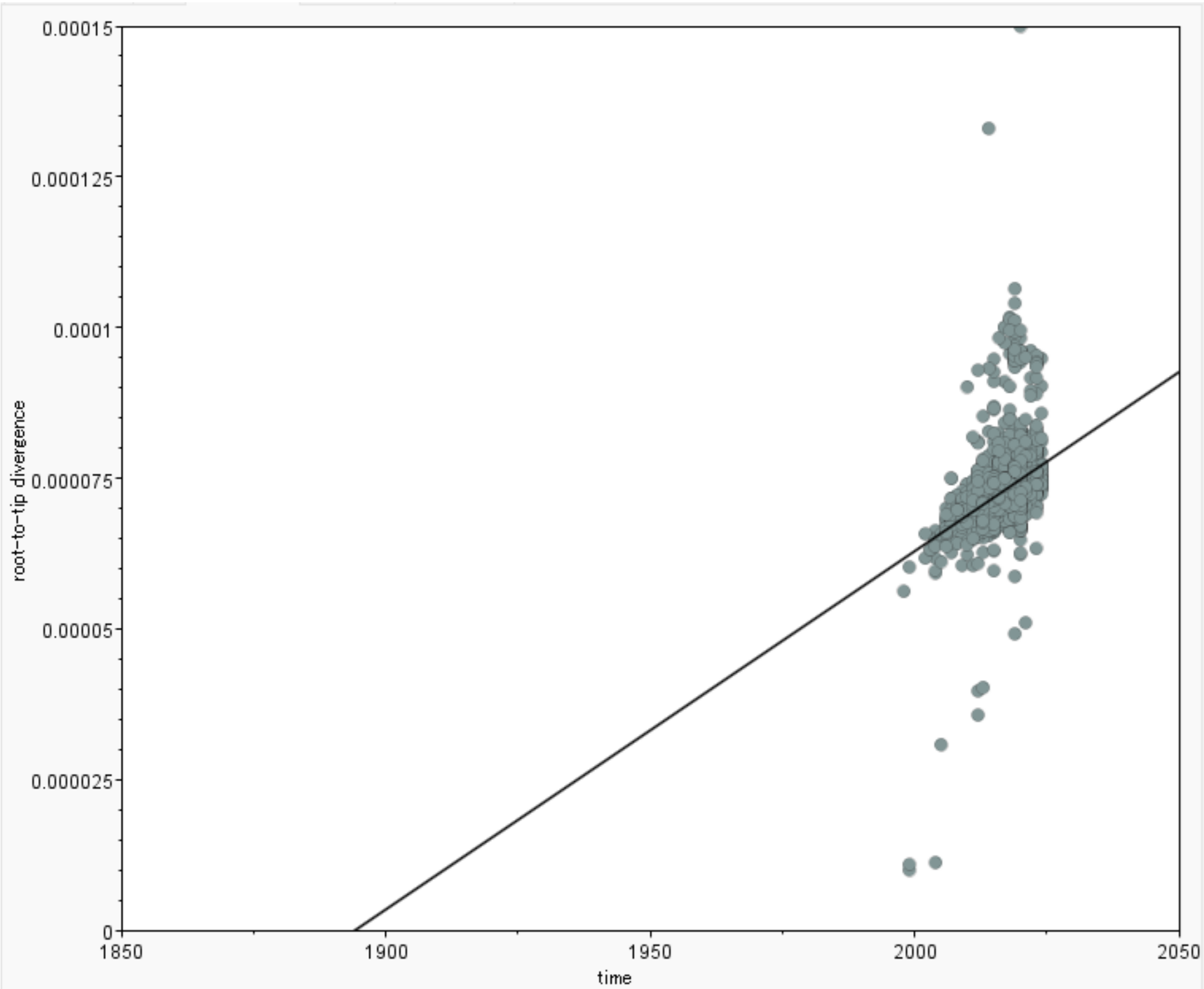

Figure S3. Root-to-tip regression analysis of temporal signal in ST131-A genomes.

Root-to-tip genetic distances were regressed against sampling years using TempEst on the recombination-filtered maximum-likelihood phylogeny of 2,905 ST131-A genomes after excluding the clade B outgroup. The best-fitting root was selected by maximising R^2. The analysis showed a modest but positive temporal signal (R^2 = 0.160; correlation coefficient = 0.400; slope = 5.94×10^{-7} substitutions/site/year).

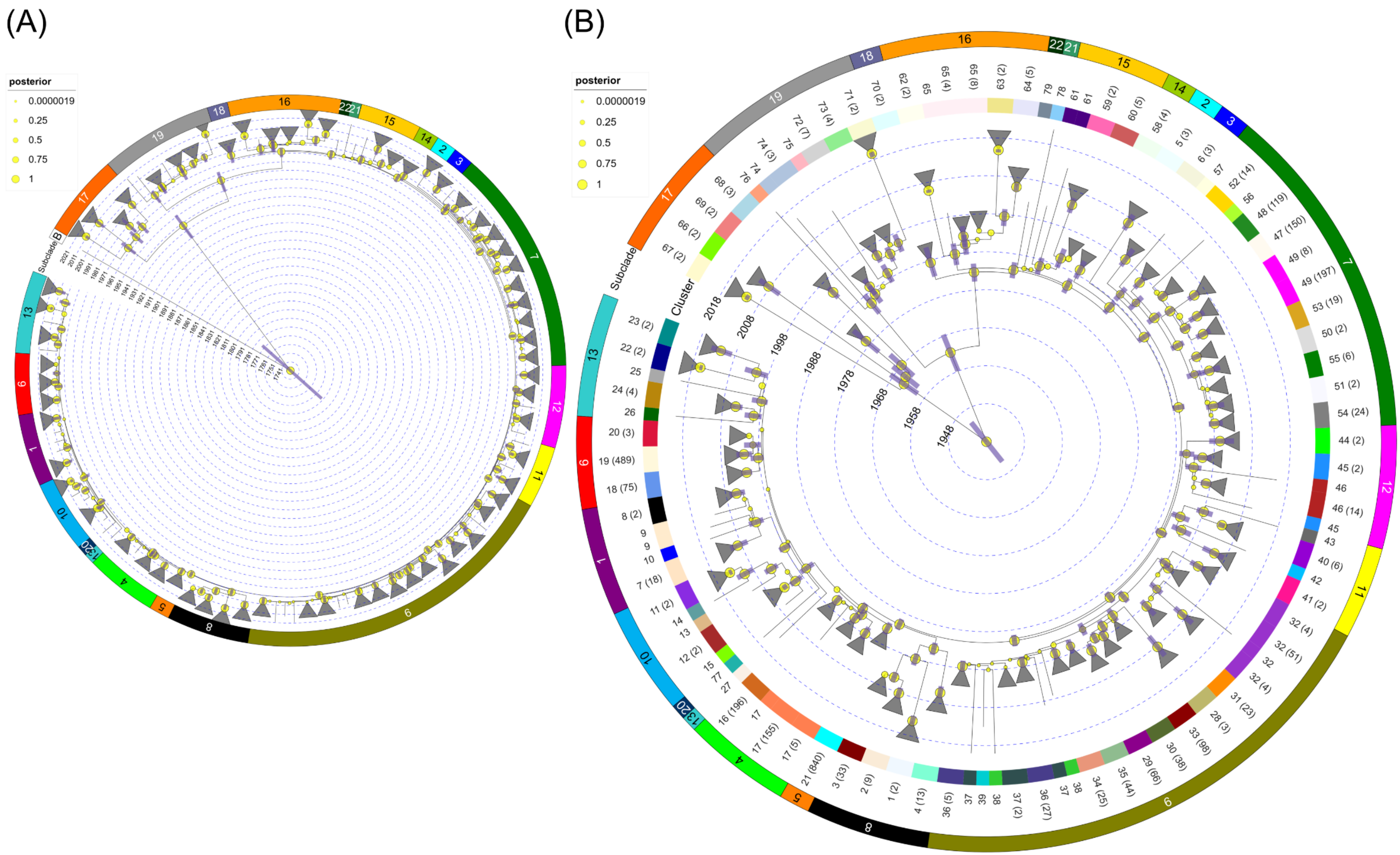

Figure S4. Time-scaled maximum clade credibility tree of ST131 clade A with collapsed clusters under the strict molecular clock model.

(a) Overview of the complete tree including the clade B outgroup. Numbers in the subclade color strip denote A1–A22; the prefix "A" is omitted for clarity. (b) Enlarged view of the ST131-A portion of the same tree after pruning the clade B outgroup for visualisation. Non-monophyletic clusters were collapsed separately by monophyletic component, with the number of genomes represented by each collapsed component shown in parentheses.

Horizontal node bars indicate the 95% highest posterior density intervals for estimated node ages. Yellow circles indicate posterior probabilities, with circle size proportional to posterior support.

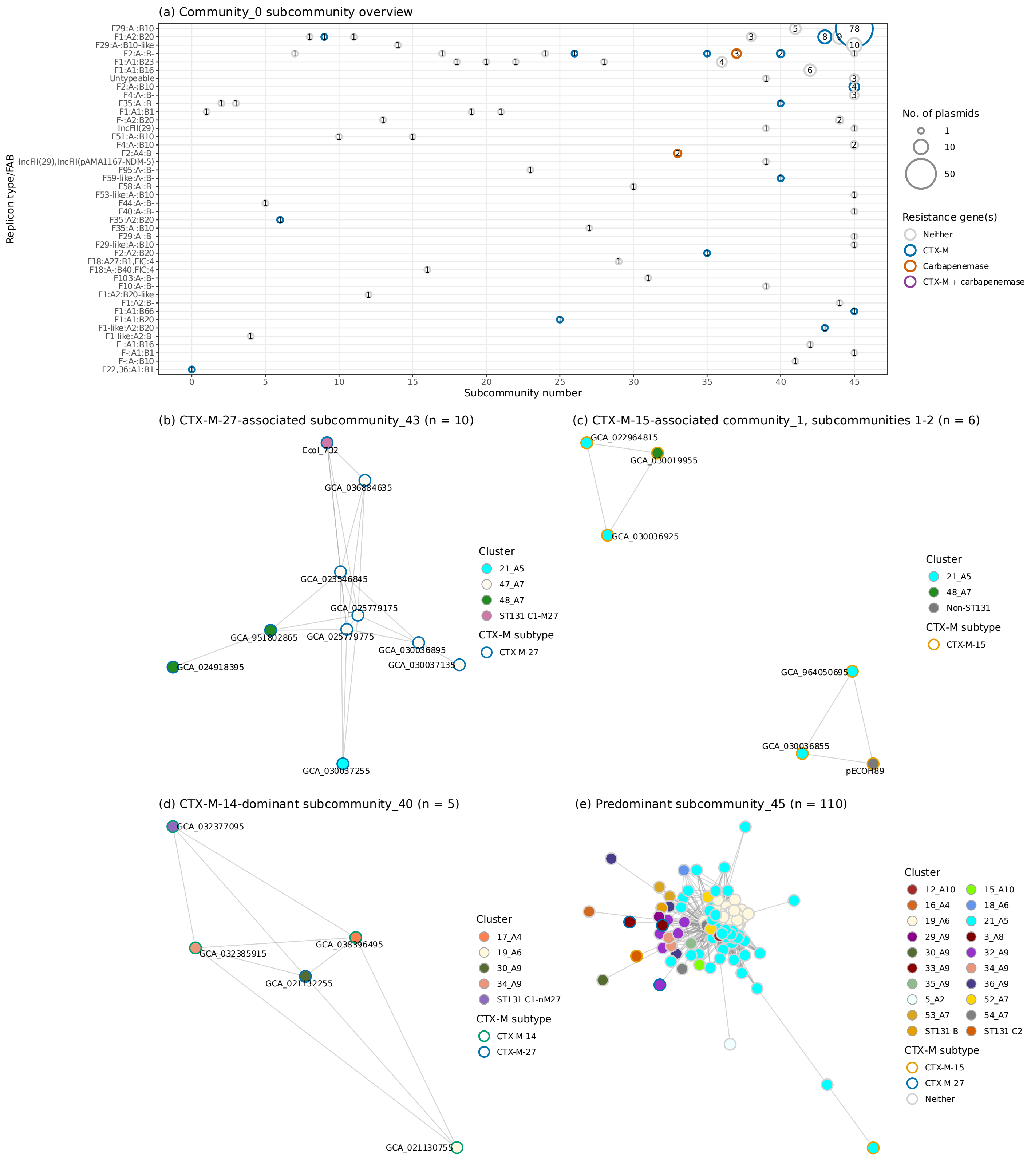

Figure S5. Pling-based relationships among plasmids from ST131-A complete genomes.

(a) Overview of the subcommunities within the largest Pling community (community_0) among ST131-A plasmids. Each circle represents one subcommunity and is positioned according to its subcommunity number and replicon type/FAB profile. The x-axis indicates the subcommunity number. Circle size and the number inside each circle indicate the number of plasmids in the subcommunity. Circle outline indicates whether the subcommunity contained at least one plasmid carrying a CTX-M gene, a carbapenemase, both, or neither.

(b) Network of CTX-M-27-associated subcommunity_43 within community_0, comprising F1:A2:B20 or F1-like:A2:B20 plasmids. This panel additionally includes a reference F1:A2:B20 plasmid from Ecol_732 (clade C1-M27).

(c) Network of CTX-M-15-associated subcommunities 1 and 2 within community_1, comprising IncFIB(H89-PhagePlasmid) plasmids. This panel additionally includes a reference IncFIB(H89-PhagePlasmid) plasmid from pECOH89 (*E. coli* non-ST131).

(d) Network of CTX-M-14/27-associated subcommunity_40 within community_0, comprising F2:A-:B-, F35:A-:B-, or F59-like:A-:B- plasmids. This panel additionally includes a reference F2:A-:B- plasmid from GCA_032377095 (host strain TUM2802; clade C1-non-M27).

(e) Network of subcommunity_45, the largest subcommunity within community_0, comprising mainly F29:A-:B10 and related plasmids. This panel additionally includes a reference F29:A-:B10 plasmid from SaT040 (clade B) and an F2:A1:B- plasmid from EC958 (clade C2).

In panels (b)–(e), each node represents a complete plasmid, node fill indicates the host cluster, and node outline indicates the detected resistance-gene category. Edges represent plasmid relationships identified by Pling using a sequence-containment threshold of 0.5 and a DCJ-indel distance cutoff of 4. Network layout and edge lengths are provided for visualisation and do not represent phylogenetic relationships or evolutionary distances. Node labels indicate the corresponding host genome or reference plasmid.
